# An unusual metal-bound 4-fluorothreonine transaldolase from *Streptomyces* sp. MA37 catalyses promiscuous transaldol reactions

**DOI:** 10.1007/s00253-020-10497-z

**Published:** 2020-03-06

**Authors:** Linrui Wu, Ming Him Tong, Andrea Raab, Qing Fang, Shan Wang, Kwaku Kyeremeh, Yi Yu, Hai Deng

**Affiliations:** 1 Department of Chemistry, University of Aberdeen, Aberdeen, AB24 3UE UK; 2 Department of Chemistry, University of Ghana, P.O. Box LG56, Legon-Accra, Ghana; 3 Key Laboratory of Combinatorial Biosynthesis and Drug Discovery (MOE) and Hubei Province Engineering and Technology Research Centre for Fluorinated Pharmaceuticals, School of Pharmaceutical Sciences, Wuhan University, Wuhan, 430071 China

**Keywords:** β-Hydroxy-α-amino acids, 4-fluorothreonine, 4-fluorothreonine transaldolase, Transaldolation, *Streptomyces* sp. MA37

## Abstract

β-Hydroxy-α-amino acids (βH-AAs) are key components of many bioactive molecules as well as exist as specialised metabolites. Among these βH-AAs, 4-fluorothreonine (4-FT) is the only naturally occurring fluorinated AA discovered thus far. Here we report overexpression and biochemical characterisation of 4-fluorothreonine transaldolase from *Streptomyces* sp. MA37 (FTaseMA), a homologue of FTase previously identified in the biosynthesis of 4-FT in *S. cattleya*. FTaseMA displays considerable substrate plasticity to generate 4-FT as well as other β-hydroxy-α-amino acids with various functionalities at C4 position, giving the prospect of new chemo-enzymatic applications. The enzyme has a hybrid of two catalytic domains, serine hydroxymethyltransferase (S) and aldolase (A). Site-directed mutagenesis allowed the identification of the key residues of FTases, suggesting that the active site of A domain has a historical reminiscent feature in metal-dependent aldolases. Elemental analysis demonstrated that FTaseMA is indeed a Zn^2+^-dependent enzyme, the first example of pyridoxal phosphate (PLP) enzyme family fused with a metal-binding domain carrying out a distinct catalytic role. Finally, FTaseMA showed divergent evolutionary origin with other PLP dependent enzymes.

**Electronic supplementary material:**

The online version of this article (10.1007/s00253-020-10497-z) contains supplementary material, which is available to authorized users.

## Introduction

**Fig. 1:**
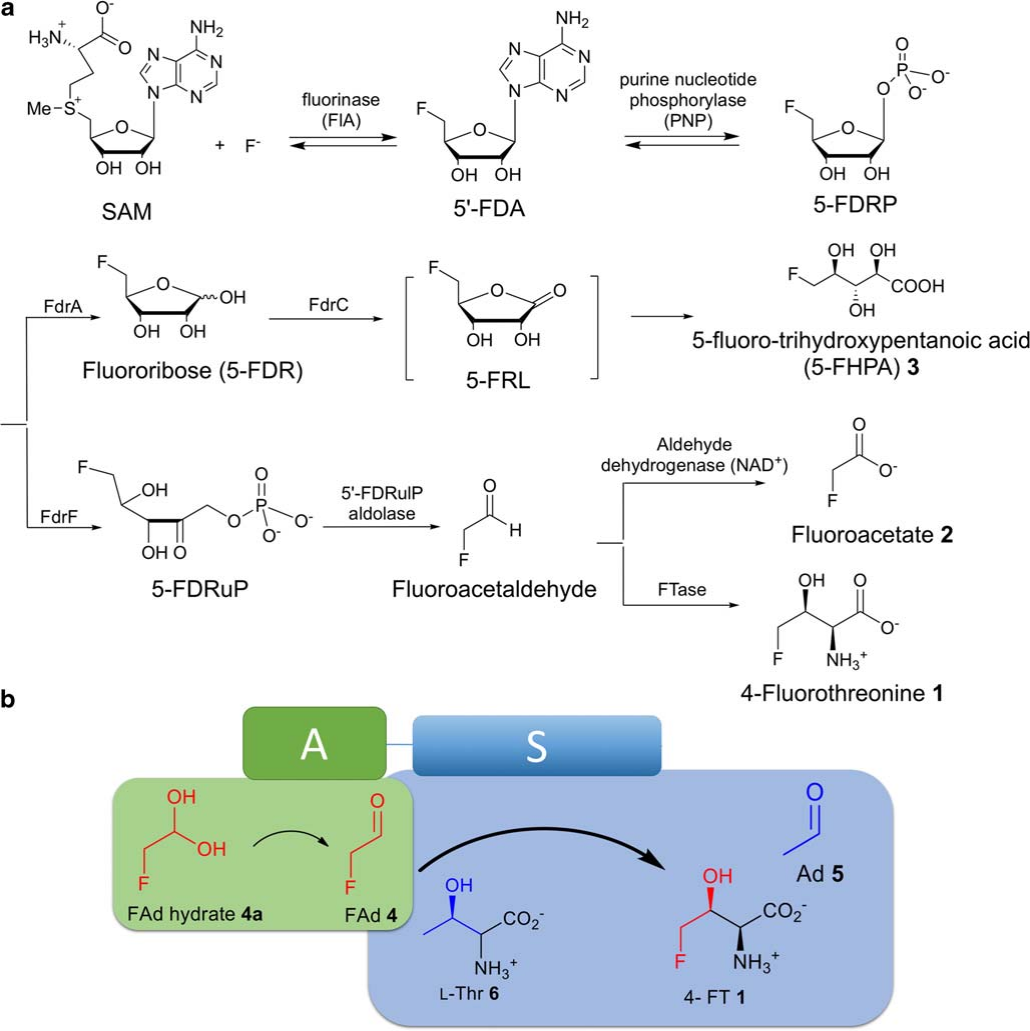
**a** The proposed biosynthetic pathway for fluorometabolites identified from *Streptomyces sp.* MA37, 4-fluorothreonine (4-FT) (**1**), fluoroacetate (4-FAd) (**2**) and FHPA (**3**). **b** A proposed model of the chemical pathway from L-threonine (L-Thr) **6** and fluoroacetaldehyde (FAd) **4** to generate **1** and acetaldehyde (Ad) **5**. The SHMT (S) domain is indicated in blue blocks, and the aldolase domain (A) is indicated as green blocks

Naturally occurring β-hydroxy-α-amino acid residues (βH-AAs) have been discovered to play important roles as key residues in many bioactive molecules (Jung and Jung 1989). Some of βH-AAs also exist as specialised metabolites (Scott et al. 2017). Among these βH-AAs, 4-fluorothreonine (4-FT) **1** is the only naturally occurring fluorinated amino acid. **1** was found to be a specialised metabolite in 1986 from the soil bacterium, *Streptomyces cattleya*, co-produced with toxic fluoroacetate **2** (Sanada et al. 1986) (Fig. 1A). Since the last decade, the biosynthetic pathway of **1** in *S. cattleya* has been established as shown in Fig. 1 (O’Hagan and Deng 2014). The hallmark of this biofluorination research is the discovery of the first native fluorination enzyme, fluorinase from *S. cattleya*, that convert *S*-adenosyl-L-methionine and fluoride ion to generate 5′-fluoro-5′-deoxy-adenosine (5′-FDA) and L-methionine (Deng et al. 2004; O’Hagan et al. 2002). Subsequently, the fluorinase has been extensively utilised as biocatalyst in generating fluorinated molecule, fluorosalinosporamide (Eustáquio et al. 2010) and radiolabelled ligands for positron emission tomography (PET) (Lowe et al. 2018; Thompson et al. 2014; Zhang et al. 2016). 4-Fluorothreonine transaldolase (FTase) is the last enzyme in the pathway that catalyses the biotransformation of fluoroacetaldehyde (FAd) **4** and L-threonine to **1** and acetaldehyde **5** (Fig. 1B) (Murphy et al. 2001). The enzyme appeared to be a hybrid protein containing two catalytic domains: the larger one (ca 440 amino acid residues in length) that is most closely related to the pyridoxal phosphate (PLP)-dependent serine hydroxymethyl transferases (SHMTs) (S domain) and the smaller one that resembles an aldolase/epimerase (ca. 145 amino acid residues in length) (A domain), connected by a short amino acid linker (~ 35 amino acid in length) (Fig. 1B) (Deng et al. 2008). Knockout of *flFT* in *S*. *cattleya* abolished the production of **1** (Zhao et al. 2012), validating the direct involvement of the *flFT* gene in the biosynthesis of 4-FT. Heterologous expression in *S. lividans* TK24 allowed the successful de novo fluorination biotransformation from inorganic fluoride ion to 4-FT **1** in vitro (Deng et al. 2008). Since then, detailed biochemical characterisation of this enzyme has yet been reproduced. More recently, McMurry et al. showed that, although 4-FT can be efficiently incorporated into protein in place of L-threonine, *S. cattleya* is evolved to manage the 4-FT toxicity by recruiting two proteins, FthB and FthC, a *trans*-acting aminoacyl-transfer RNA deacylase and a 4-FT transporter, respectively. These two proteins are highly conserved in the identified 4-FT biosynthetic locus (Mcmurry and Chang 2017).

In our metabolic profiling program to discover novel natural products from Ghanaian isolates, we have identified a talented soil bacterium, *Streptomyces* sp. MA37, which has the capacity of producing several structurally unique bioactive metabolites (Huang et al. 2015a; Huang et al. 2015b; Maglangit et al. 2019). Of particular interest here is that the strain is able to produce a range of fluorinated metabolites, including two known metabolites, **1** and **2** (Deng et al. 2014), and a new fluorinated compound, 5-fluoro-hydroxypentanoic acid (5-FHPA) **3** (Fig. 1a) (Ma et al. 2015). Genome analysis of MA37 allowed identification of the homologous biosynthetic gene clusters (BGC) of **1** and **2** compared to the one in *S. cattleya* and a new BGC that direct the synthesis of **3** with biochemical evidence that **3** is derived from 5′-fluoro-5′-deoxy-ribose-1-phosphate (5′-FDRP), the key branched intermediate of both pathways (Ma et al. 2015). Interestingly, the *flFT* gene homologue in MA37 appears to be in close proximity of the fluorinase gene, a different genetic arrangement from the Spencer cluster in *S. cattleya* (Huang et al. 2006). Herein we report biochemical characterisation of the recombinant FTase from *Streptomyces* sp. MA37 (FTaseMA). FTaseMA was overexpressed in *S. lividans* 66 for biochemical evaluation. The substrate tolerance of FTaseMA was also observed as various new β-hydroxy-α-amino acids were generated in the enzymatic assays with the corresponding substrates. The key residues of this FTaseMA were further explored using site-directed mutagenesis based on homologue modelling of two domains with two different characterised enzymes, SHMTs and aldolases, respectively. Elemental analysis demonstrated that zinc divalent ions are tightly bound in the enzyme with the stoichiometric ratio of 1:1. Taken together, a mechanistic model of the action of FTaseMA was proposed. Finally, detailed phylogenetic analysis of FTase has shed light on the evolutionary origins of FTase homologues, threonine transaldolase (TTAs), SHMTs and threonine aldolases (TAs).

## Materials and methods

### Fermentation conditions

A list of acronyms appeared in this manuscript can be found in the supporting information (Table S6). *Escherichia coli* strains were grown in Luria-Bertani (LB) broth (1% tryptone, 0.5% yeast extract, 0.5% NaCl) or LB agar (1.5% agar) at 37 °C, supplemented with the corresponding antibiotics. *E. coli* DH10B was used as the routine cloning strain for DNA manipulations. *E. coli* ET12567 was a DNA methylation deficiency stain; it serves as a host for *E. coli*-*Streptomyces* conjugation with the help of plasmid pUZ8002. *S. lividans* 66 was the model strain used for protein purification in this study. The strain containing the construct with the *flFT* gene (MBC001) was grown in MS agar media (2% soya flour, 2% mannitol, 2% agar) for spores production. The spores of MBC001 was inoculated into YEME medium (0.5% tryptone, yeast extract 0.3%, malt extract 0.3%, glucose 1%, sucrose 10.3%) and grown for 48 h (28 °C and 180 rpm), supplemented with 50 μg/mL apramycin for protein overexpression. Protein overexpression was induced by addition of thiostrepton (50 μg/mL, final concentration) in the culture with further cultivation (28 °C and 180 rpm) for 72 h.

### Genomic DNA extraction

The genomic DNA in this study was extracted from 2-mL cell culture. Cell pellet was harvested by centrifugation and resuspended in 500 μL SET buffer. The cell suspension was mixed with lysozyme (4 mg/mL, final concentration) and incubated at 37 °C for 30 min. SDS (60 μL, 10% (w/v))and NaCl (200 μL, 5 M) was then added to the mixture, followed by another incubation at 60 °C for 30 min. The protein was precipitated with the mixture of phenol, chloroform and isoamylol (500 μL, ratio of 25:24:1), and the resultant mixture was mixed by vortex. The supernatant was separated by centrifuge and transferred to a new Eppendorf tube with isopropanol for DNA precipitation (0.8 volumes). The precipitated DNA was washed with 75% (v/v) ethanol, followed by the second wash of 100% ethanol. DNA pellet was dried at room temperature and dissolved in sterile Milli-Q water (200 μL).

### Plasmid construction

To generate the recombinant plasmid for N-His_6_ FTaseMA overexpression, for *E. coli* hosted overexpression, a codon-optimised *flFT* gene (GenBank accession No. MN481988) was synthesised by GenScript, USA. For *Streptomyces* hosted overexpression, the PCR product *flFT* was amplified using the MA37 genomic DNA as the template, followed by subcloning onto the linearized *Streptomyces* protein overexpression vector pGM1190 (*Nde*I, *Hin*dIII), which contains an inductive *tipA* promoter, to produce pMBC001. To express the site-mutated FTaseMA, pMBC002, pMBC003, pMBC004, pMBC005 and pMBC006 were constructed with two arms flanking the mutation site based on the vector pMBC001 (*Nde*I, *Hin*dIII) (Table S2–3).

### Protein expression and purification

A single colony of *S. lividans* 66 exoconjugate isolated from *E. coli-Streptomyces* conjugation plate after PCR confirmation was streaked onto MS plate supplemented with apramycin (50 μg/mL) and incubated at 28 °C for 10 days. Spores were collected from the plate and inoculated into YEME (500 mL) medium containing apramycin (50 μg/mL) (28 °C, 180 rpm). Thiostrepton (50 μg/mL) was added to the cell culture 2 days after the inoculation, followed by another 3 days cultivation (28 °C, 180 rpm). Cell pellets were collected by centrifuge (4600 rpm, 10 min) and resuspended in loading buffer (50 mM Tris-HCl, 300 mM NaCl, pH 7.2). The cell resuspension was sonicated, and the lysate was centrifuged (13,300 rpm, 10 min) to remove cell debris residue. The supernatants were then passed through a syringe filter (0.22-μm pore size) before loaded onto a His-Bind affinity column (3-mL bed volume). The column was washed with loading buffer (5 column-volume (CV)), and the recombinant protein was eluted by gradient imidazole elution buffer (10–500 mM imidazole), and the fractions containing FTaseMA were pooled together. After desalting and buffer exchange with the washing buffer (50 mM Tris-HCl, 50 mM NaCl), the semi-purified protein was loaded onto Bio-Scale™ Mini UNOsphere™ Q Cartridge (Bio-Rad, 5 mL column volume). The target protein was eluted by gradient NaCl concentration (50 to 500 mM). Finally, the fractions containing FTaseMA was loaded on an ENrich™ High-Resolution Size Exclusion Column (Bio-Rad, 25 mL) with isocratic elusion. The resulting purified protein was concentrated with Amicon Ultra-4 concentrators (Millipore, 10 kDa cut-off) and preserved under − 80 °C. The fractions and the purity of the recombinant protein was analysed with Bis-Tris SDS-PAGE. Protein concentration was determined using Colibri Microvolume Spectrometer at A_280_ (molar absorption coefficient, 1.5).

### Standard FTaseMA enzyme assay

The standard assays were performed with fluoroacetaldehyde (2 mM), L-threonine (5 mM), PLP (0.1 mM) and Tris-HCl buffer (50 mM, pH 7.5) and incubated at 28 °C. The reaction was quenched by heat (100 °C, 10 min). The negative control was employed with the same condition except for using denatured enzyme (100 °C, 10 min). After centrifugation (14,000 ×*g*, 5 min), the supernatant was mixed with 0.5 volumes of D_2_O and subjected to ^19^F-NMR analysis (400 Hz, 512 scans).

### Kinetic analysis of FTaseMA assay

The kinetic analysis of FTaseMA assay was performed using fluoroacetaldehyde and L-threonine as substrates. Each assay contained 50 mM Tris-HCl buffer (pH 7.5), PLP (0.1 mM), FTaseMA (14 μM), fluoroacetaldehyde (5 mM) and L-threonine (0.5, 1, 2, 5, 10, 20 mM) in a final volume of 100 μL. The reaction was initiated by addition of threonine to the system. The reaction was finally quenched by adding 2 volumes of perchloric acid (3 M). The reactions were performed in triplicate. A HPLC analysis using DNPH as derivatization agent was employed to monitor the generation of acetaldehyde after the transaldolation process. The standard curve of acetaldehyde DNPH derivatives was established (Fig. S5). The concentration of the aldehyde generated from the enzymatic reactions was estimated against the standard curve. Michaelis-Menten kinetics were then calculated. In all cases, the rates were calibrated with the background noises. For the kinetics analysis of various acetaldehyde derivatives, varied concentrations of the aldehyde substrates (0.5–20 mM) were used while keeping the concentration of L-Thr constant (5 mM). For the kinetics analysis of L-*allo*-threonine (0.5, 1, 2, 5, 10, 20 mM), the concentration of fluoroacetaldehyde was kept constant (5 mM). The same reaction condition and derivatization method were also applied.

### Synthesis of fluoroacetaldehyde

Fluoroacetaldehyde in this study was synthesised according to the method in literature (Moss et al. 2001). 2-Fluoroethanol (1 mL 17.0 mmol) was added to pyridinium dichromate (PDC, 2.00 g, 5.3 mmol) in dichloromethane (30 mL). The reaction mixture was heated (60 °C) under reflux for overnight, followed by distillation into a separating funnel containing Milli-Q water (5 mL). The aqueous layer from the solution was analysed using ^19^F-NMR.

### HR-ESI-MS procedures

High-Resolution Electrospray Ionisation Mass Spectrometry (HR-ESI-MS) was determined using LC MS Thermo Scientific MS system (LTQ Orbitrap) coupled to a Thermo Instrument HPLC system (Accela PDA detector, Accela PDA autosampler and Accela Pump, C18 SunFire 150 × 46 mm Waters®). The following parameters were used: capillary voltage 45 V, capillary temperature 320 °C, auxiliary gas flow rate 10–20 arbitrary units, sheath gas flow rate 40–50 arbitrary units, spray voltage 4.5 kV, and mass range 100–2000 amu (maximum resolution 30,000). Additionally the samples were run on an Agilent 6200 series TOF/6500 series Q-TOF instrument with a scan rate of 1.5 Hz, scan range from 100 to 1000, variable CID energy, 3.5 kV source voltage, fragmentor 175 V (± 200%) and reference masses (121.05087 and 922.00979) enabled. The instruments were optimised as required.

### DNPH derivatization

The reaction mixtures containing aldehyde compounds from enzymatic reactions were mixed with perchloric acid (3 M, 2 volumes) to denature the proteins, followed by neutralisation with sodium acetate solution (3 M, 2 volumes, pH 9.0). The supernatant was separated from protein precipitate by centrifuge (13,330 rpm, 10 min, 4 °C) and then was mixed with DNPH solution pre-dissolved in 6 N HCl (20 mg/mL, 3 volumes). The resultant mixtures were incubated (30 °C, 180 rpm, 1 h) before quenched with sodium acetate solution (3 M, 3 volumes pH 9.0). The derivatized aldehyde-DNPH products were extracted with acetonitrile (3 volumes). The organic layer was separated, followed by evaporation using nitrogen dryer to obtain the dried aldehyde-DNPH products.

### Marfey’s derivatization

Amino acids generated in the enzymatic reactions were subjected to derivatization according to Marfey’s method (Bhushan and Brückner 2004). The enzymatic reaction mixtures were quenched with of chloroform (0.5 volumes). The aqueous solution was dried using nitrogen dryer. The dried residue was redissolved in a mixture solution (100 μL) of triethylamine/methanol/water (1:1:2). The resultant mixture was mixed with 1-fluoro-2-4-dinitrophenyl-5-L-alanine amide (FDAA) solution in acetone (18.4 mM) (1:1 v/v). The derivatization was conducted in the dark (40 °C, 90 rpm, 1 h), followed by evaporation using nitrogen dryer. The dried material was finally redissolved in methanol (500 μL) for HPLC and LC-MS.

### Elemental analysis using HPLC coupled with inductively coupled plasma mass spectrometer (ICP-MS)

The protein solution (140 μM) was first centrifuged using an Amicon Ultra-4 concentrators (Millipore, 10 kDa cut-off) to remove any elements that were not associated with the protein and concentrated. To confirm that all sulphur and zinc present in the solution was indeed associated with the protein, size exclusion chromatography (Superdex 75 10/300, GE Healthcare Life Science, UK) using the buffer system (0.1 M Tris-HCl buffer, pH 7.4) was carried out. The HPLC system was Agilent 1290 system with autosampler cooled to 4 °C coupling with quaternary pump and DAD detector. The protein sample (20 μL) was injected to the column after the column was equilibrated with the buffer (0.1 M Tris-HCl buffer, pH 7.4) (1 mL/min). The outlet of the DAD was directly connected to an Agilent 8800 ICP-MS/MS, which was run using O_2_ as reaction gas and monitoring the following isotopes: ^32^S- > ^48^SO, ^64^Zn, ^67^Zn. The same set-up was used to determine the influence of EDTA on zinc bound to the protein. To determine the ratio of zinc to sulphur, the protein solution was diluted appropriately with 1% (v/v) nitric acid for quantitative sulphur and zinc analysis. To calculate the enzyme, zinc ratio, the amount of sulphur determined by total element analysis was divided by the amount of zinc. The results 19 ± 1.0 equals within the precision of the method, the number of sulphur containing amino acids (*n* = 19), cysteine and methionine, are presented in the enzyme (Table S5).

### Quantification and statistical analysis

The Michaelis-Menten kinetics parameters for FTaseMA assays were calculated using the Solver module on Excel, Microsoft, based on non-linear regression method. The enzymatic reactions were performed in triplicate, and all rates were calibrated with the background noise in HPLC.

### Spectroscopic measurements of protein-bound PLP concentrations

All UV-visible spectra in this study were recorded on a Colibri Microvolume Spectrometer (Titertek-Berthold). For the quantification of enzyme-bound PLP, the enzyme solution was denatured (100 °C, 10 min) followed by centrifugation to obtain the supernatant. The concentration of PLP in the supernatant was calculated based on the standard curve of PLP measured in UV-Vis (388 nm). Prior to enzymatic assays, FTaseMA was first incubated with PLP (1 mM). Excess PLP was removed from the protein solution by exchanging into Tris-HCl buffer (50 mM, pH 7.5) containing 100 mM NaCl using Amicon Ultra-4 concentrators (Millipore, 10-kDa cut-off) at 4 °C. The resultant protein was then concentrated to 93 μM using an Amicon column (10-kDa MWCO). For the UV-Vis assays, FTaseMA were incubated with different concentrations of L-threonine (0–50 mM) on ice (30 min) before the spectra were recorded.

### Phylogenetic analysis

The sequence used to construct the phylogenetic tree was obtained from the National Centre for Biotechnology Information (NCBI) (Benson et al. 2008) database and the Protein Data Bank (PDB) (Bernstein et al. 1977). Sequences were initially aligned using ClustalW by MEGA7 (Kumar et al. 2018) with default parameters. The phylogenetic tree was built by MEGA7 using maximum likelihood method with default parameters.

## Results

### Overexpression of 4-FTaseMA

Although FTase activity has been known since 2001 (Murphy et al. 2001), this group of enzymes has not been biochemically characterised in details. It has become our research objective to express the *flFT* (GenBank accession No. HG428740.1) homologous gene from *Streptomyces sp.* MA37 in different hosts. Firstly, we chose *E. coli* to express this gene. To this end, a codon-optimised synthetic gene was first cloned into various expression vectors, followed by transformation into *E. coli* BL21 (DE3). We only observed soluble recombinant proteins in the *E. coli* strain with the pCold-TF vector harbouring the *flFT* gene as confirmed in SDS-PAGE analysis (Fig. S1). To assay the activity of the recombinant protein, FAd **4** was then synthesised according to literature (Moss et al. 2001). However, incubation of either cell-free extract or purified soluble proteins with L-threonine (1 mM), fluoroacetaldehyde (~ 1 mM) and PLP (10 μM) results in no production of 4-FT **1**, indicating that this recombinant protein is not functional.

**Fig. 2:**
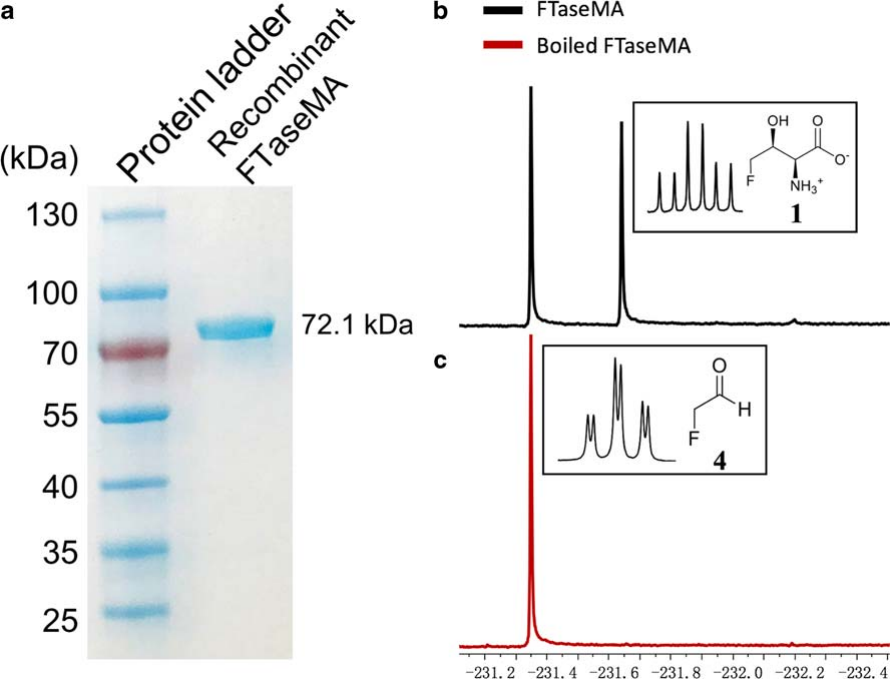
Overexpression of FTaseMA and enzymatic assays. **a** The SDS-PAGE analysis of FTaseMA. **b** The ^19^F-NMR analysis of FTaseMA enzyme assay with a inset of ^19^F-NMR coupling spectrum of 4-FT **1**(*dt*, δ_F_-231.67, ^2^*J*_*HF*_ 9.3 Hz, ^3^*J*_*HF*_ 48.0 Hz). **c** The negative control when using denatured FTaseMA with a inset of ^19^F-NMR coupling spectrum of FAc **4** (*dt*, δ_F_-231.31, ^2^*J*_*HF*_ 20.0 Hz, ^3^*J*_*HF*_ 48.0 Hz)

We then cloned this gene into the shuttle vector, pGM1190 (Muth et al. 1989), followed by overexpression in three *Streptomyces* hosts (*S. lividans* TK24, *S. lividans* 66 and *S. albus* J1074) in our laboratory, a common strategy that has been used to overexpress recombinant proteins in *Streptomyces* hosts. Incubation of the cell-free extracts of these recombinant *Streptomyces* strains with appropriate substrates and cofactors gave rise to the 4-FT production as monitored by ^19^F-NMR. The cell free extract from the *S. lividans* 66 recombinant variant gave the highest production of **1** (Fig. S2) among these three recombinant variants, while the least activity was found in *S. lividans* TK24 recombinant strain (Fig. S2). Thus the *S. lividans* 66 recombinant variant was used for further protein overexpression. The recombinant protein was purified to near homogeneity (Fig. 2a) as a bright yellow protein with the estimated yield of 2-mg/L culture, through three chromatographic methods. The native protein appeared to be a dimer based on the estimation of the retention time of the size exclusion chromatography, consistent with the previous report (Murphy et al. 2001). The purified protein was also confirmed by MS-MS sequencing. The purified recombinant protein appears a bright yellow colour, and the stoichiometry between bound PLP and the protein was estimated to be 1:1 (Fig. S3). The recombinant protein has absorbance maxima at 335 and 425 nm, in which the enolimine peak at 325 nm is dominant (Fig. S4a). However, the UV-Vis spectra of the protein incubate with various concentrations of L-threonine led to the dominant maxima absorbance value at 425 nm, suggesting the formation of the external aldimine, similar to the previous report (Raman et al. 2009) (Fig. S4c). Incubation of the purified recombinant enzyme with L-threonine **6** (1 mM), **4** (~ 1 mM) and PLP (10 μM) resulted in the production of **1** (Fig. 2b). In the control assay with boiled FTaseMA, the formation of **1** was not observed (Fig. 2c).

### Biochemical analysis of FTaseMA in generating 4-FT 1

^19^F-NMR is a convenient and efficient analytical method to directly measure the production of **1** and other fluorometabolites. However, ^19^F-NMR lacks sensitivity which makes it difficult to be used in enzymatic kinetic studies (Qin et al. 2012). Although a direct and sensitive fluorine-specific method of high-resolution continuous source molecular absorption spectrometry has been developed (Qin et al. 2013), this method requires standard curves of both standard **1** and **4** to calculate the concentrations. Neither **1** nor **4** are commercially available or can be prepared synthetically as pure compounds.

**Fig. 3:**
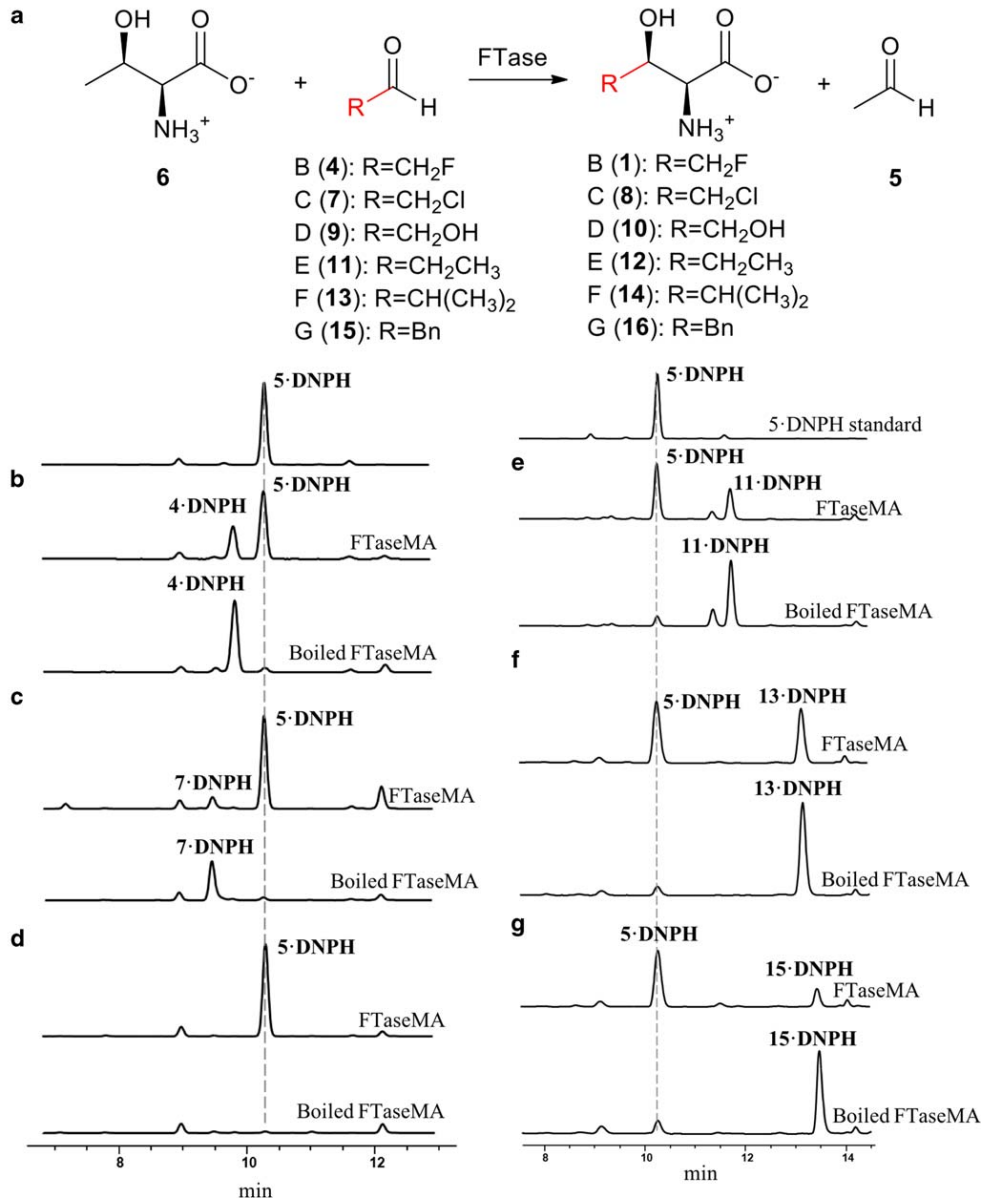
HPLC analysis of the products generated in the FTaseMA assay. Fluoroacetaldehyde **4** (B), chloroacetaldehyde **7** (C), glycolaldehyde **9** (D), propanal **11** (E), isobutyraldehyde **13** (F) and phenylacetaldehyde **15** (G) was used as substrates in the assays. The L-threonine derivatives generated from the assay were confirmed by ESI-HRMS

To circumvent this, we developed a derivatization method and monitored the acetaldehyde production by HPLC. Brady’s reagent (2, 4-dinitrophenylhydrazine (DNPH)) has been widely used as derivatization agents of carbonyl functional groups associated with ketones and aldehydes to form hydrazone adducts (Guan et al. 2012). We first established the standard curve of the commercially available acetaldehyde adducts (**5**-DNPH) (Fig. S5). Then we performed the enzymatic reactions, followed by the derivatization of the formed acetaldehyde for the discontinuous HPLC-based analysis (Fig. 3). The identities of aldehyde adducts of **4** and **5** were confirmed by high-resolution mass spectra (HRMS) (Fig. S11, Table S4). We also derivatized **1** and **6** using Marfey’s reagent for improved retention time and detection by LC-HRMS.

The kinetic parameters of this enzyme were determined from non-linear regression. The assays were conducted using a range of L–Thr concentrations (0.5–20 mM), while the FAd concentration was kept constant at 5 mM, and then a range of FAd **4** (0.12–2.4 mM), while the L–Thr was held at 20 mM. The apparent *K*_m_ value for FAd **4** was calculated to be 0.24 mM (Table 1). The apparent **K**_m_ value for L–Thr was 1.18 mM, indicating a lower affinity for the enzyme.

**Table 1:** Kinetic parameters for the activity of FTase towards various amino acids and aldehydes

Substrate	*k*_cat_ (min^−1^)	*K*_M_ (mM)	*k*_cat_/ *K*_M_ (M^−1^ min^−1^)
Fluoroacetaldehyde (**4**)	1.799 ± 0.019	0.241 ± 0.002	7473 ± 81
Chloroacetaldehyde (**7**)	1.425 ± 0.011	1.350 ± 0.001	1056 ± 9
Glycolaldehyde (**9**)	1.466 ± 0.053	0.357 ± 0.010	4096 ± 148
Propanal (**11**)	1.213 ± 0.047	1.453 ± 0.179	841 ± 70
Isobutyraldehyde (**13**)	0.808 ± 0.045	0.535 ± 0.078	1525 ± 129
Phenylacetaldehyde (**15**)	0.585 ± 0.008	0.358 ± 0.006	1635 ± 23
L-threonine (**6**)	3.131 ± 0.056	1.180 ± 0.007	2654 ± 47
L-*allo*-threonine (**19**)	0.423 ± 0.031	1.180 ± 0.006	360 ± 26

### FTaseMA displays considerable substrate plasticity

With recombinant FTaseMA available, we then set out to probe the substrate plasticity using various amino acids while keeping **4** at the constant concentration (5 mM). Overnight incubation of the purified enzyme (14 μM), PLP and FAd (5 mM) with D-*allo*-threonine, D-threonine or glycine or alanine or leucine (10 mM) led to no production of **1**, indicating that these amino acids are not the substrates of FTaseMA, while the assay with L-serine generated a very small amount of **1** as observed in ^19^F-NMR (Fig. S8). To our surprise, enzyme assay with L-*allo*-threonine **19** led to produce **1** albeit with less reactivity towards **19** compared to L-threonine (Fig. S9).

Next, we used commercially available acetaldehyde analogues, such as chloroacetaldehyde (ClAd) **7**, glycolaldehyde (GlAd) **9**, propanal (PpAd) **11**, isobutyraldehyde (*i*-butylAd) **13**, phenylacetaldehyde (PhAd) **15**, trifluoroacetaldehyde (TFA) **17**, trifluoropropanal **18** and 3-fluoropropanal **20**, while keeping L–Thr (5 mM) constant. Interestingly, the enzyme can accept the aldehyde analogues as substrates with less efficient *k*_cat_ values (1.43 min^−1^ for ClAd **7**, 1.47 min^−1^ for GlAd **9**, 1.21 min^−1^ for PpAd **11**, 0.81 min^−1^ for **13** and 0.58 min^−1^ for **15**) compared with the one for FAd **5** (Fig. S10). The identities of the corresponding amino acid products, 4-chlorothreonine **8**, 4-hydroxyl-threonine **10**, 4-methyl-threonine **12**, 4-isobutyl-threonine **14** and 4-phenyl-threonine **16**, were confirmed by ESI-HRMS analyses of the Marfey’s adducts generated from the enzymatic mixtures (Fig. S11). TFA and trifluoropropanal are not substrates of the enzyme.

### Probing the active site of FTaseMA

FTases are distinct from classical TTAs in that the enzymes use FAd **4** as a substrate. FAd **4** is heavily hydrated in water (Cobb et al. 2004). The enzymes may evolve to accelerate the transformation of hydrate to increase the aldehyde availability for the transaldolation. Interestingly, FTases have two putative domains, SHMT/TTA (S) domain, which presumably handle the transaldol reaction, and the aldolase/epimerase (A) domain that may catalyse the dehydration reaction of FAd hydrate.

Structural prediction (Kelley et al. 2015) of the A domain suggested good structural similarity with the metal-dependent class II aldolases, 5-deoxyribulose 1-phosphate aldolase (DrdA, PDB accession No. 6BTG) from *Bacillus thuringiensis* (Beaudoin et al. 2018) and 5-methylthioribulose-1-phosphate dehydratase (MtnB, PDB accession No. 4M6R) in the methionine salvage pathway (Kang et al. 2014), despite low sequence identity (24% and 20%, respectively). DrdA and MtnB catalyse the cleavage of ribulose-1-phosphate derivatives to dihydroxyacetone phosphate (DHAP) and acetaldehyde derivatives in reversible fashion. DrdA is one of the key enzymes in the salvage pathway of 5-deoxyribose, a toxic by-product of the radical *S*-adenosyl-L-methionine (SAM) enzyme (Beaudoin et al. 2018), and MtnB is involved in the methionine pathway (Kang et al. 2014). These class II aldolases normally contain an active site divalent metal acting as the Lewis acid type catalyst, coordinated by three conserved histidine residues (His95-His97-His157 in DrdA). The general mechanism of these aldolases involves a conserved active site glutamate residue (Glu76 in DrdA) that directs proton transfer of the aldol to generate an enediol intermediate, coordinated by the bound metal. Homology modelling (Kelley et al. 2015) of the S domain of FTaseMA revealed a resemblance with a SHMT from the hyperthermophile archaea, *Methanocaldococcus jannaschii* (Angelucci et al. 2014). The lysine residue (Lys237 in the S domain of FTaseMA) is highly conserved in all active sites of SHMTs.

Examination of the predicted structure of the aldolase/epimerase domain of FTaseMA suggested a catalytic tetrad, Glu573-His551-His553-His598, with the essential residue of Asn484 (Kang et al. 2014) that may position Glu573 residue towards the active site through hydrogen bonds, based on the structural and primary amino acid alignments (Fig. S13). It was hypothesised that this catalytic tetrad would direct the proton transfer of the diol of FAd hydrate to generate the aldehydic or enolic form, coordinated by a divalent metal ion. Indeed, elemental analysis of FTaseMA demonstrated that the stoichiometry of the bound zinc and the enzyme was estimated to be 1:1 (Fig. S6, Table S5), suggesting that this is the first example of metal-binding PLP-dependent enzymes (Du and Ryan 2019). Zinc ions appear to be strongly associated with FTaseMA. Addition of EDTA into FTaseMA results in no inhibition of the activities, and our elemental analysis demonstrated that Zn^2+^ ions are still bound in FTaseMA after EDTA treatment (Fig. S7).

Nonetheless, to determine the roles of the identified residues, site-directed mutagenesis (SDM) was carried out, the mutated plasmids pMBC002–006 were generated based on pMBC001. Mutations of Lys237 to Ala, His551 to Ala, His598 to Ala and Asn484 to Ala significantly reduced the relative specific activity to around 13.1%, 14.5%, 18.1% and 27.7%, respectively, suggesting that these residues are important to the biological activity. Changing Glu573 to Ala, however, resulted in the instability of the purified recombinant protein which prevented further biological evaluation. Nonetheless, the important roles of the corresponding Glu residues in other metal-dependent class II aldolases have been highlighted by mutagenesis experiments.

## Discussion

Naturally occurring β-hydroxy-α-amino acids (βH-AAs) are key residues in many bioactive metabolites, such as glycopeptide antibiotics (Williams 1984), cyclopeptides (Genet 1996) and β-lactone antibiotic obafluorin (Schaffer et al. 2017; Scott et al. 2017). Some of βH-AAs are also specialised metabolites with a range of antimicrobial and anticancer bioactivities. Among these βH-AAs, 4-fluorothreonine **1** is the only fluorinated β-hydroxy-α-amino acid natural product. It is known that fluorinated α-amino acids constitute an important class of compounds due to their unique metabolic and physiological activities. They have been applied as biological tracers, enzyme inhibitors and for medical applications (Salwiczek et al. 2012). Selective fluorination to generate fluorinated amino acids has been well-developed. However, only few reported enzymatic syntheses of fluorinated amino acids. In 2007, Steinreiber et al. examined L-threonine aldolase (L-TA) from *Pseudomonas putida* and D-threonine aldolase (D-TA) from *Alcaligenes xylosoxidans* in the biotransformation of generating various 3-alkylserine derivatives. Incubating fluoroacetaldehyde with glycine in the presence of L-TA and PLP gave 4-FT (L-*syn*-fluorothreonine) with 50% yield, while D-TA was able to generate D-*syn*-fluorothreonine in the presence of PLP, manganese ion and DTA together with glycine and fluoroacetaldehyde (Steinreiber et al. 2007).

FTases are the only naturally occurring enzymes that catalyse the unique transaldolation reaction between L-threonine and fluoroacetaldehyde to generate 4-FT. The activities of FTase was initially identified in *S. cattleya*, and the transaldolation mechanism of FTase was deduced from isotopic labelling studies in 2001 (Murphy et al. 2001). Subsequently, the full-length *flFT* gene was reported, and the recombinant FTase was successfully overexpressed in *S. lividans* TK24 (Deng et al. 2008). Since then, it has been our research objective to biochemically characterise this group of enzymes for potential biotransformation. FTaseMA shares high homology (80% aa identity) with the original FTase from *S. cattleya*. We found that FTaseMA displays considerable substrate promiscuity to generate 4-fluorothreonine as well as β-hydroxy-α-amino acids using various aldehydes and L-threonine as substrates. β-Hydroxy-α-amino acids are key building blocks and important biomolecules (Delle Monache et al. 1997; Fanning et al. 2005; Hibi et al. 2015; Pirrung et al. 1993; Steinreiber et al. 2007), suggesting that FTaseMA could be used as a potential versatile biocatalyst. Strikingly, the enzyme can also utilise L-*allo*-threonine as substrates albeit less efficient than the natural substrate L-threonine, a clear difference compared to other L-threonine transaldolases that catalyse the similar transaldol reaction to FTases. These L-TTAs display narrow substrate specificity towards **6** and cannot utilise L-*allo*-threonine as substrates (Barnard-Britson et al. 2012). This can be exemplified by two biochemically characterised L-TTAs: the L-threonine, uridine-5′-aldehyde transaldolase, LipK, in the biosynthesis of lipopeptidyl nucleoside natural product (Barnard-Britson et al. 2012), and the L-threonine, 4-nitrophenylacetaldehyde transaldolase, ObaG in the biosynthesis of obafluorin (Schaffer et al. 2017; Scott et al. 2017).

FTase homologous genes have been mainly found in actinomycetes (Mcmurry and Chang 2017). In 2019, a new FTase homologue (GenBank Accession No. VFJ60022) emerged in NCBI from the Gram-negative bacterium, *Candidatus Kentron* sp. DK, a symbiont of the marine ciliate, *Kentrophoros* (Seah et al. 2019). However, a comparative genomic and a sequence similarity network analyses using the sequences of FTases, FthB and FthC as sequence queries suggested that neither the *flA* gene homologue nor *fth*B and *fth*C homologous genes were found in close proximity of the *flFT* opening reading frame (*orf*) or in the draft genome of *Candidatus Kentron* sp. DK (Fig. S14). Thus, it is less clear if there is any metabolic significance for this *flFT* gene or if there is any benefit for the bacterial communities in the corresponding symbiotic host, the marine ciliate *Kentrophoros*.

The key difference between FTases and other L-TTAs is that FTases contain two domains, S domain to handle the transaldol reaction and A domain to transform FAd hydrate to aldehydic form. Homologue-modelling guided site-directed mutagenesis that demonstrated four key residues in A domain of FTaseMA play an important role of biological activity of FTaseMA, strongly suggesting that A domain of FTaseMA may have evolved specifically for the dehydration reaction from a historical activity of other aldolases. MEME prediction (Ma et al. 2014) of the amino acid sequences among this group of FTases allowed the identification of conserved amino acid residues, Lys237, Asn484, His551, Glu573 and H598 in FTaseMA, consistent with our SDM results (Fig. S15). Our phylogenetic analysis (Kumar et al. 2018) also suggested that FTases, with both domains (Fig. S15) or with the S domain only (Fig. S16), formed a distinct clade different from the classical L-TTAs, L-TAs and SHMTs. Elemental analysis demonstrated that FTaseMA is a metal-bound PLP-dependent enzyme, and the stoichiometry between FTaseMA and the divalent metal, zinc, is 1:1. This is the first example of PLP-dependent enzyme family that hybridizes with a metal-dependent aldolase performing a distinct catalytic function.

**Scheme 1:**
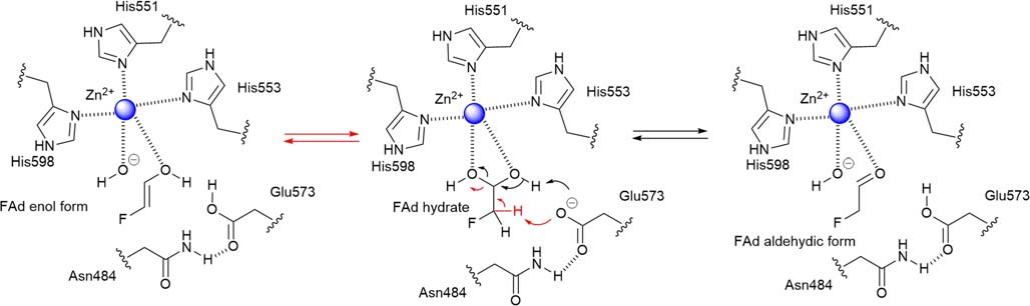
A proposed model of the dehydrate process catalysed by the A domain of FTaseMA. The pathway in black (right) to form FAd aldehydic form and the one in red (left) to form FAd enolic form which will then be transferred to the SHMT domain for transaldolation reaction.

A proposed model of the dehydration is illustrated in Scheme 1. The Glu573 may act as a general base to abstract the hydrogen atom at one of the diol groups of FAd hydrates to form FAd aldehydic from. Depending on the position of Glu573, one cannot exclude that Glu573 may abstract one of the acidic hydrogens at C2 position of FAd hydrates to form enol intermediate. Nonetheless, both pathways are coordinated by Zn^2+^ to stabilise the transition state of the transform for the subsequent transaldolation reaction in the SHMT/L-TTA-like domain.

In summary, we have biochemically characterised FTaseMA, the last committed enzyme in the 4-FT pathway from *Streptomyces* sp. MA37. The enzyme also displays good substrate promiscuity to generate various β-hydroxy-α-amino acids. FTaseMA is the first example of metal-bound PLP-dependent enzymes as observed in our elemental analysis and homologue modelling-guided site-directed mutagenesis. Phylogenetic investigation revealed that FTases form their own discrete evolutionary lineage separated from L-TTAs and even more distinct from L-TAs and SHMTs (Fig. S15 and S16).

## Electronic supplementary material

ESM 1 (PDF 3177 kb)
