# Supplementary material for: An unusual metal-bound 4-fluorothreonine transaldolase from Streptomyces sp. MA37 catalyses promiscuous transaldol reactions
Source: Appl Microbiol Biotechnol. 2020 Mar 6;104(9):3885–96. doi: 10.1007/s00253-020-10497-z (PMC7162832; doi:10.1007/s00253-020-10497-z)
Supplement: Supplementary file 1 — (PDF 3177 kb) [file 253_2020_10497_MOESM1_ESM.pdf]

Applied Microbiology and Biotechnology

An unusual metal-bound 4-fluorothreonine transaldolase from *Streptomyces* sp. MA37 catalyses promiscuous transaldol reactions

Linrui Wu[1], Ming Him Tong[1], Andrea Raab[1], Qing Fang[1], Shan Wang[1], Kwaku Kyeremeh[2], Yi Yu[3], Hai Deng[1]

1. Department of Chemistry, University of Aberdeen, Aberdeen AB24 3UE, UK
2. Department of Chemistry, University of Ghana, P.O. Box LG56, Legon-Accra, Ghana
3. Key Laboratory of Combinatorial Biosynthesis and Drug Discovery (MOE) and Hubei Province Engineering and Technology Research Centre for Fluorinated Pharmaceuticals, School of Pharmaceutical Sciences, Wuhan University, Wuhan 430071, China

*Corresponding author: Dr Hai Deng, Tel: (+44) 01224 272953; Fax: (+44) 01224 954742
E-mail address: h.deng@abdn.ac.uk

Table S1. List of numbered compounds

**Number**	**Compounds**
**1**	4-Fluorothreonine
**1a**	Derivatization adduct of **1**
**2**	Fluoroacetate
**3**	5-FPHA
**4**	Fluoroacetaldehyde
**4a**	Derivatization adduct of **4**
**5**	Acetaldehyde
**5a**	Derivatization adduct of **5**
**6**	L-threonine
**6a**	Derivatization adduct of **6**
**7**	Chloroacetaldehyde
**7a**	Derivatization adduct of **7**
**8**	4-Chlorothreonine
**8a**	Derivatization adduct of **8**
**9**	Glycolacetaldehyde
**9a**	Derivatization adduct of **9**
**10**	4-Hydroxyl-threonine
**10a**	Derivatization adduct of **10**
**11**	Propanal
**11a**	Derivatization adduct of **11**
**12**	4-Methyl-threonine
**12a**	Derivatization adduct of **12**
**13**	Isobutyraldehyde
**13a**	Derivatization adduct of **13**
**14**	4-Isobutyl-threonine
**14a**	Derivatization adduct of **14**
**15**	Phenylacetaldehyde
**15a**	Derivatization adduct of **15**
**16**	4-Phenyl-threonine
**16a**	Derivatization adduct of **16**
**17**	Trifluoroacetaldehyde
**18**	Trifluoropropanal
**19**	L-*allo*-threonine
**20**	3-fluoropropanal

Table S2. The primer sequences used in this study

Primers	Sequence (5’-3’)	Application
pMBC001-F	tgccgcgcggcagcCATATGGTGCCACCCGAAGCCGTTACCGGCA	pMBC001 cloning
pMBC001-R	tcgagtgcggccgcAAGCTTTCACCGTCGGCGCACCGCCCGGCGG	
FTaseM-F	TGCCGCGCGGCAGCCATATGCCACCCGAAGCCGTTACCGG	Universal mutation generation
FTaseM-R	CGAGTGCGGCCGCAAGCTTTCACCGTCGGCGCACCGCCCG	
pMBC002-R	GGGCCGAAGAGCGTCGCGTGGGTGTTGGCCTGGAGGATGT	pMBC002 cloning
pMBC002-F	CACGCGACGCTCTTCGGCCCGCAGAAGG	
pMBC003-R	TCCGTACAGCTGATCGCGCCCGCCGAGTCGATCTGGTGGG	pMBC003 cloning
pMBC003-F	CGCGATCAGCTGTACGGACGCGGACGA	
pMBC004-R	TGATGCAGTGGTTCGCCACGATGTACGCGGCGCCGGTCCT	pMBC004 cloning
pMBC004-F	AGGCGTACGGCAGCGTCGCACTGGCCGA	
pMBC005-F	GGGCCGAAGAGCGTCGCGTGGGTGTTGGCCTGGAGGATGT	pMBC005 cloning
pMBC005-R	CTACTCCGCGACCGGGTACTGCCTGCTGGGCGCGGT	
pMBC006-F	AGAAGACGAGGCCCGCCCGGCGGACGTACAGCACCCGGCT	pMBC006 cloning
pMBC006-R1	CCGGGCGGGCCTCGTCTTCTGGGCCACCGG	
pMBC006-R2	TCCCCACGCCGGCTCGCGATATCGCCGAACCTGGCCGGGCGAGG	

Table S3. The plasmids generated in this study

Plasmids	Descriptions	
**Protein expression of FTaseMA with N-terminal 6×His tag in *Streptomyces lividans* 66, *Streptomyces lividans* TK24 and *Streptomyces albus* J-1074**		
pMBC001	Wide type FTaseMA	The PCR product of *flFT* amplified from MA37 genomic DNA with the primers, pMBC001-F and pMBC001-R (Table S1), was digested with *Nde*I and *Hin*dIII, followed by ligation into the corresponding site of pGM1190.
pMBC002	Site mutation (K237A)	The two PCR products (see primers in Table S1) of *flFT* containing the target mutation site at overlapping region were introduced into pGM1190 (*Nde*I, *Hin*dIII) by using In-fusion Cloning.
pMBC003	Site mutation (N484A)	
pMBC004	Site mutation (H551A)	
pMBC006	Site mutation (E573A)	
pMBC006	Site mutation (H598A)	
**Protein expression of FTaseMA with N-terminal 6×His tag in *E. coli* BL21 (DE3)**		
pMBC007	Codon optimized wild type FTaseMA	The codon optimized *flFT* was loaded on to expression vector pET32a
pMBC008	Codon optimized wild type FTaseMA	The DNA fragment *flFT* digested from pMBC007 with *Eco*RI and *Hin*dIII was ligated into the corresponding site of pCold-TF

Table S4. HPLC-MS Data of the Compounds in the Study

Products	Formula	calc. m/z	det. m/z	Error (ppm)
**The compounds produced in enzymatic assays**				
**1a**	$C_{13}H_{17}FN_5$	390.1056	390.1057	0.366
**8a**	$C_{13}H_{17}ClN_5$	406.0760	406.0764	0.920
**10a**	$C_{13}H_{18}N_5O_9$	388.1099	388.1102	0.609
**12a**	$C_{14}H_{20}N_5O_9$	386.1306	386.1293	1.459
**14a**	$C_{15}H_{22}N_5O_8$	400.1463	400.1465	-0.5
**16a**	$C_{19}H_{22}N_5O_8$	448.1463	448.1463	0.0
**5a**	$C_8H_7N_4O_4^-$	223.0469	223.0473	1.9
**The substrates used in enzymatic assay**				
**4a**	$C_8H_6FN_4O_4^-$	241.0379	241.0383	-2.0
**7a**	$C_8H_6ClN_4O$	257.0083	257.0079	1.8
**9a**	$C_8H_7N_4O_5^-$	239.0422	239.0422	0.1
**11a**	$C_9H_9N_4O_4^-$	237.0629	237.0629	0.0
**13a**	$C_{10}H_{13}N_4O_4$	253.0931	253.0934	0.943
**15a**	$C_{14}H_{13}N_4O_4$	301.0931	301.0931	0.0
**6a**	$C_{13}H_{18}N_5O_8$	372.1150	372.1154	0.997

Table S5. The ICP-MS determination of the ratio of the amount of sulphur to zinc in FTaseMA solution. The run was performed in triplicates. The ratio of sulphur to zinc and amount of protein in solution was calculated from amount of sulphur determined in the analysis.

	Mean	Standard deviation
S (μM)	5503	270
Zn (μM)	287	17
ratio S/Zn	19	1.0
μM protein	290	14

Table S6. The list of acronyms.

Acronyms	Full Name
4-FT	4-Fluorothreonine
F-AAs	Fluorinated Amino Acids
FAd	Fluoroacetaldehyde
5-FHPA	5-Fluoro-hydroxypentenoic Acid
PLP	Pyridoxal Phosphate
SHMT	Serine Hydroxymethyl Transferase
BGC	Biosynthetic Gene Cluster
L-TTA	Threonine Transaldolase
TA	Threonine Aldolase
DNPH	2, 4-Dinitrophenylhydrazine
HRMS	High Resolution Mass Spectra
ClAd	Chloroacetaldehyde
GlAd	Glycolacetaldehyde
PpAd	Propanal
i-butylAd	Isobutyraldehyde
PhAd	Phenylacetaldehyde
TFA	Trifluoroacetaldehyde
Cl-Thr	4-Chlorothreonine
Hydroxyl-Thr	4-Hydroxyl-threonine
Methyl-Thr	4-Methyl-threonine
Isobutyl-Thr	4-Isobutyl-threonine
Phenyl-Thr	4-Phenyl-threonine
CV	Column-Volume
HR-ESIMS	High-Resolution Electrospray Ionisation Mass Spectrometry

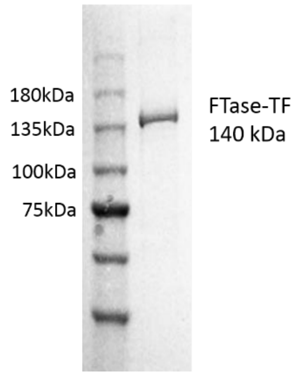

Figure S1. The SDS-PAGE gel of FTaseMA (~75 kDa) fused with TF tag (~65 kDa) purified from *E. coli* BL21 (DE3).

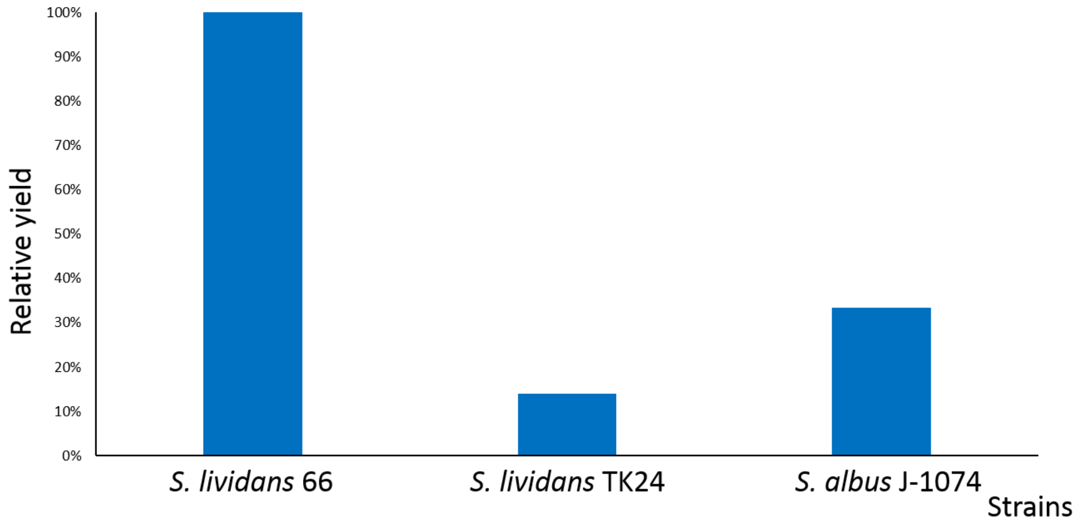

Figure S2. The relative yield of recombinant FTaseMA from *Streptomyces lividans* 66, *Streptomyces lividans* TK24 and *Streptomyces albus* J-1074. Negative control assays were conducted with empty vector pGM1190 and no transformation activity was detected.

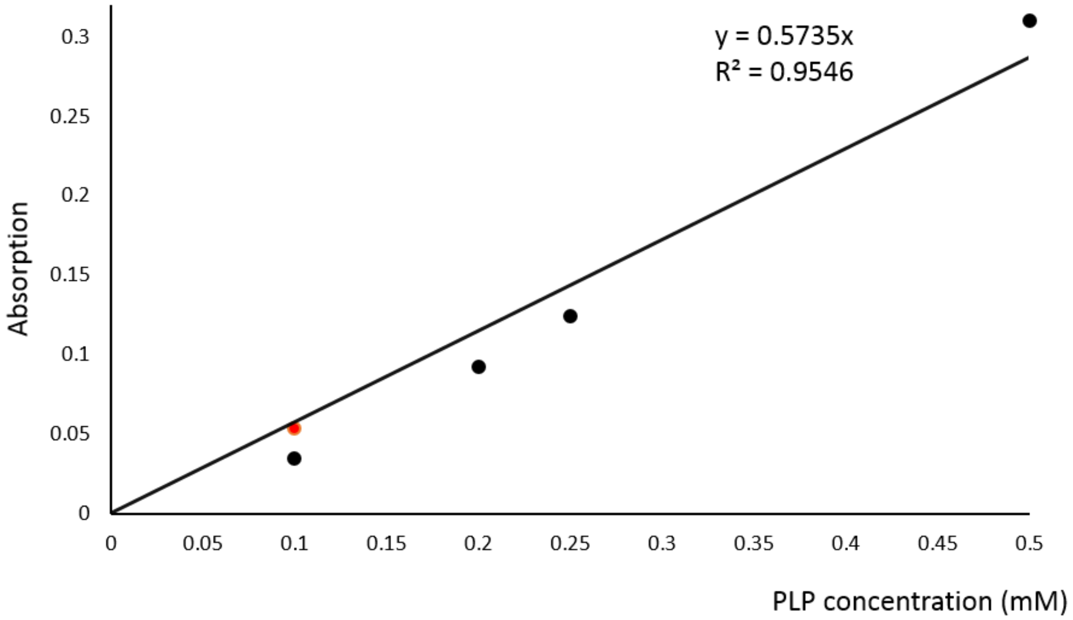

Figure S3. The standard curve for pyridoxal phosphate (PLP). The curve was established by measuring the UV-Vis absorption of gradient standard PLP solutions at 388 nm (Black dots). For the FTaseMA protein sample (93 μM), the absorption after heat-inactivation and centrifugation reads 0.054 (Red dot), demonstrating the PLP concentration in the supernatant to be 0.094 μM, confirming the stoichiometry between FTaseMA and PLP to be 1:1.

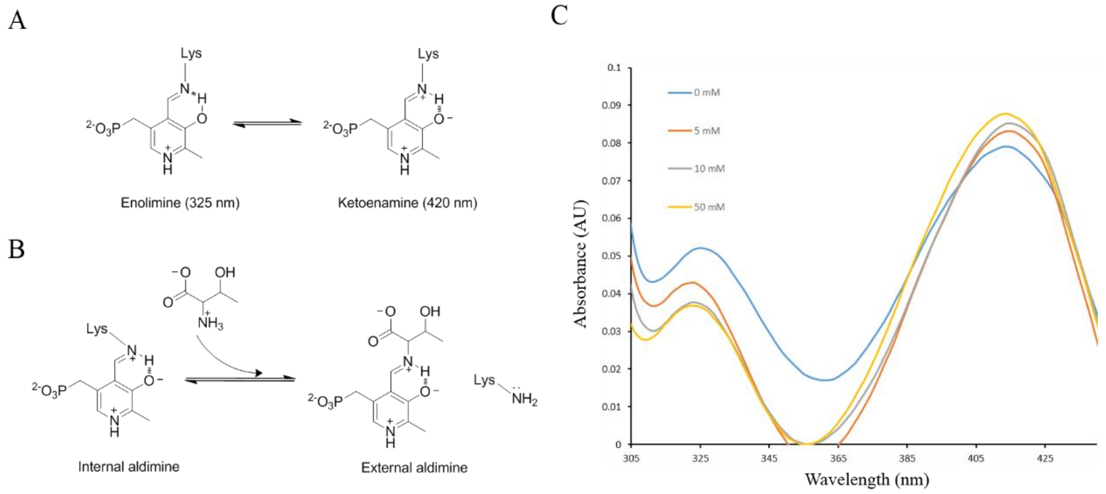

Figure S4. UV-Vis analysis indicating that FTaseMA contains protein-bound PLP. **(A)** The tautomerism between the enolimine and ketoenamine form of the PLP-lysine residue adduct. **(B)** The formation of external aldimine between PLP and L-threonine. **(C)** UV/Vis spectrum of FTaseMA exhibiting peaks at 325 nm and 420 nm, representing the enolimine and ketoenamine forms of the PLP, respectively. After incubation with gradient concentrations of L-threonine, external aldimine was formed, which led to an increase at 420 nm peak and loss of 325 nm peak.

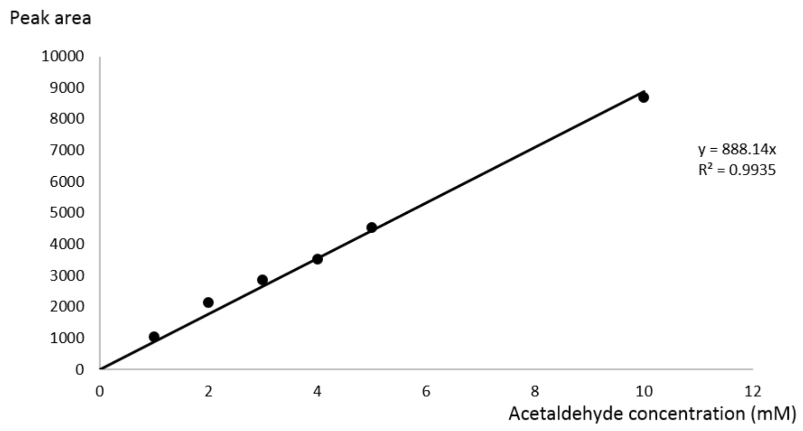

Figure S5. The standard curve of the concentrations of acetaldehyde-DNPH adducts based on HPLC peak integrations.

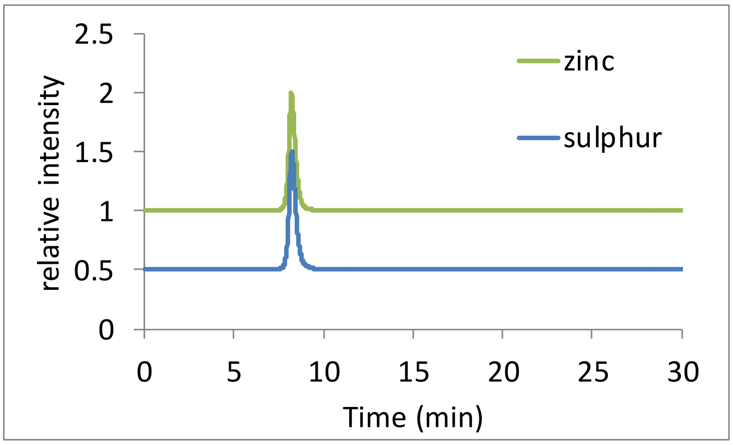

Figure S6. Separation of purified FTaseMA using size exclusion chromatography-ICP-MS/MS, separation by Superdex 75, 0.1 mol/L Tris/HCl pH 7.4. Shown are the elemental traces of sulphur and zinc. The protein was eluting at 8 min. No sulphur or zinc-containing compounds are present except in the protein signal.

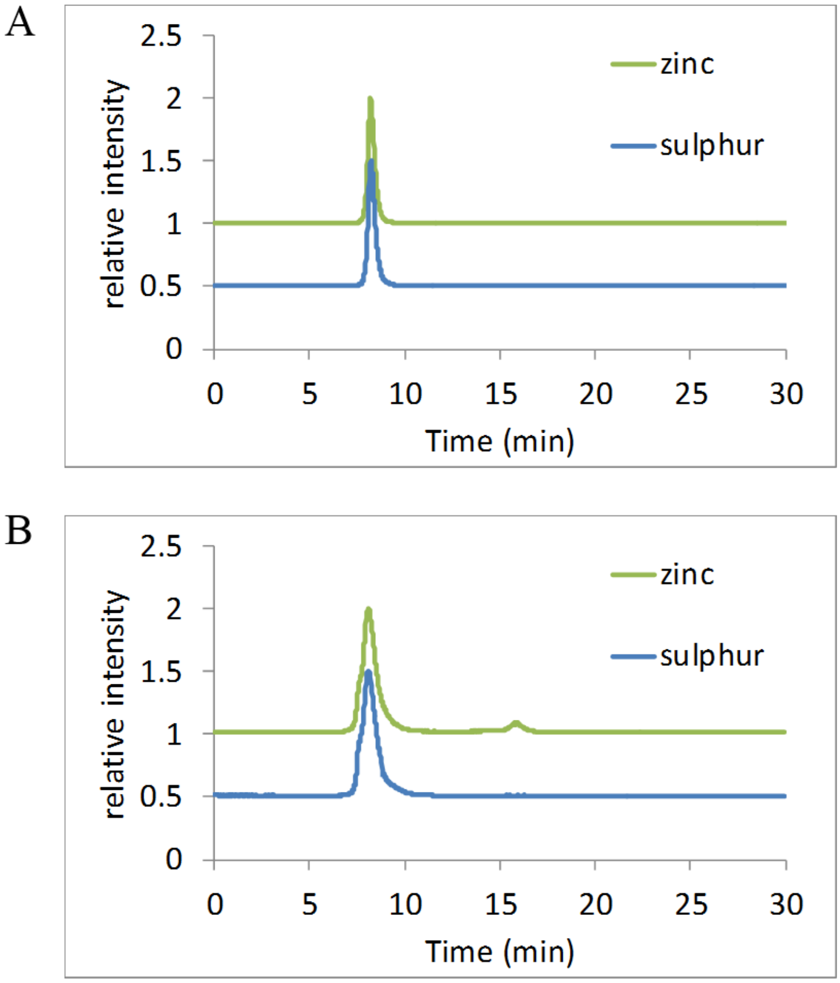

Figure S7. EDTA treatment of protein **(A)** Native FTaseMA. Shown are the elemental traces of sulphur and zinc. **(B)** FTaseMA after treatment with EDTA. The zinc/sulphur signal ratio of the protein is identical before and after the treatment of EDTA. The small zinc peak at ca. 16 min comes from zinc contamination in the column picked up by EDTA.

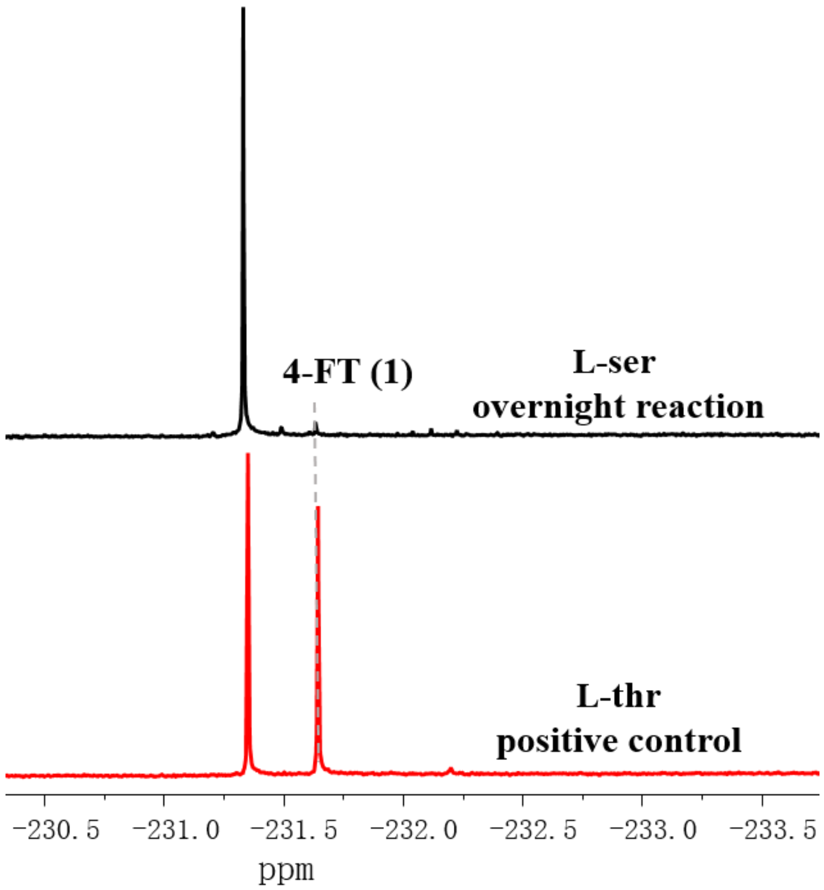

Figure S8. ^{19}F-NMR analysis of the enzymatic products. Top: Overnight incubation of FTaseMA with L-Serine, PLP and FAd **5** suggesting the production of a trace amount of 4-FT **1**. **Bottom**: Incubation of FTaseMA with L-threonine, PLP and FAd **5**.

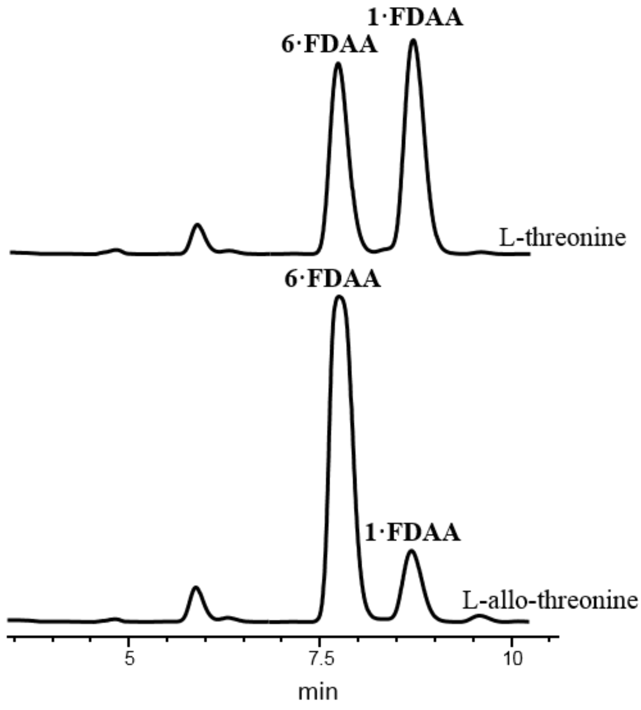

Figure S9. The HPLC analysis of the Marfey's adduct, F-Thr-FDAA, in the FTaseMA assays. Top HPLC trace: L-threonine (10 mM) as substrate. Bottom HPLC trace: L-*allo*-threonine (10 mM) as substrate.

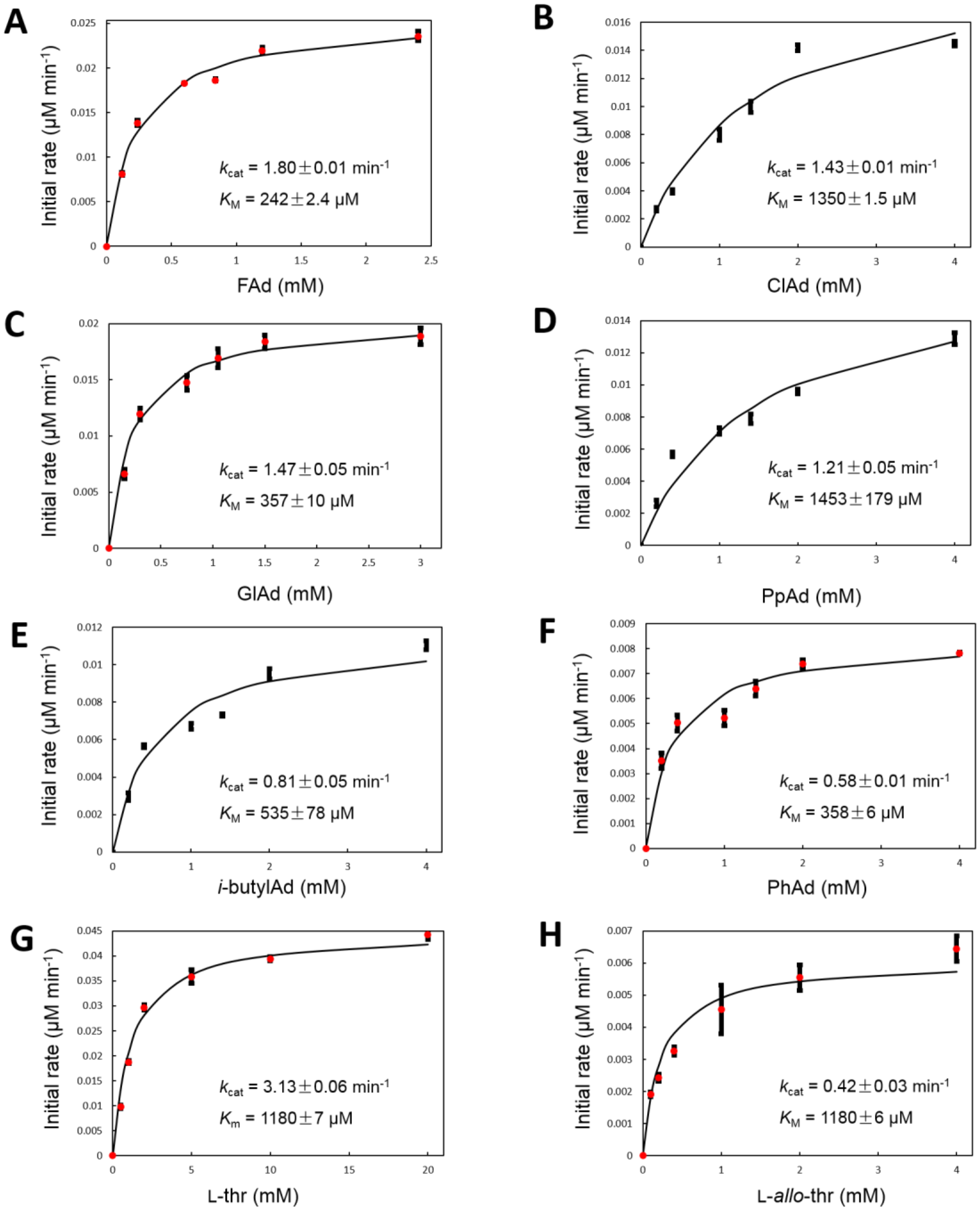

Figure S10. Michaelis-Menten curves for FTaseMA with different substrates used in the study. (A) FAd. **(B)** ClAd. **(C)** GlAd. **(D)** PpAd. **(E)** *i*-butylAd. **(F)** PhAd. **(G)** L-Thr. **(H)** L-*allo*-Thr

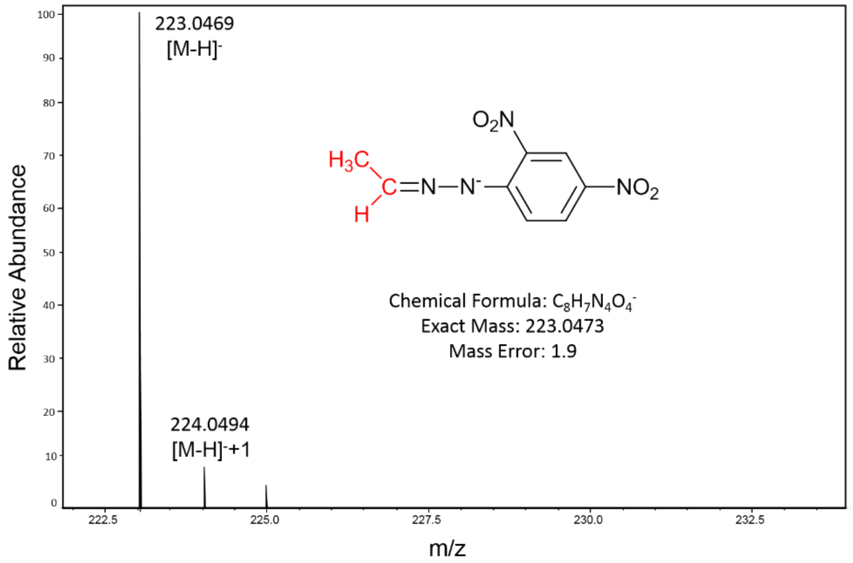

11.1. HPLC-ESI-HRMS analysis of **5a**

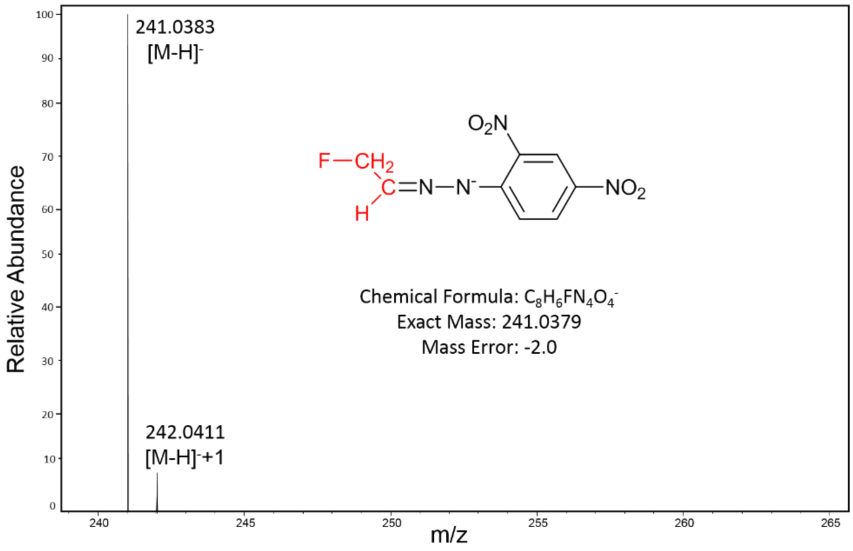

11.2. LC-QTOF analysis of **4a**

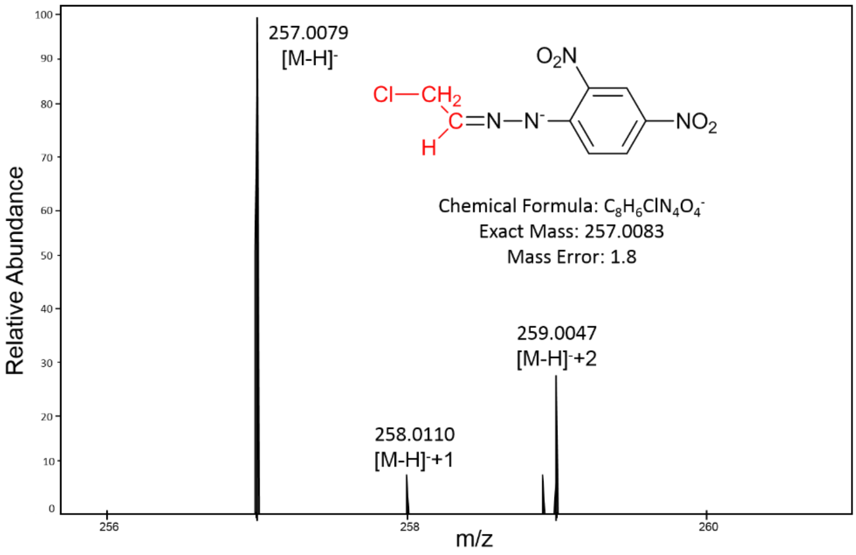

11.3. LC-QTOF analysis of **7a**

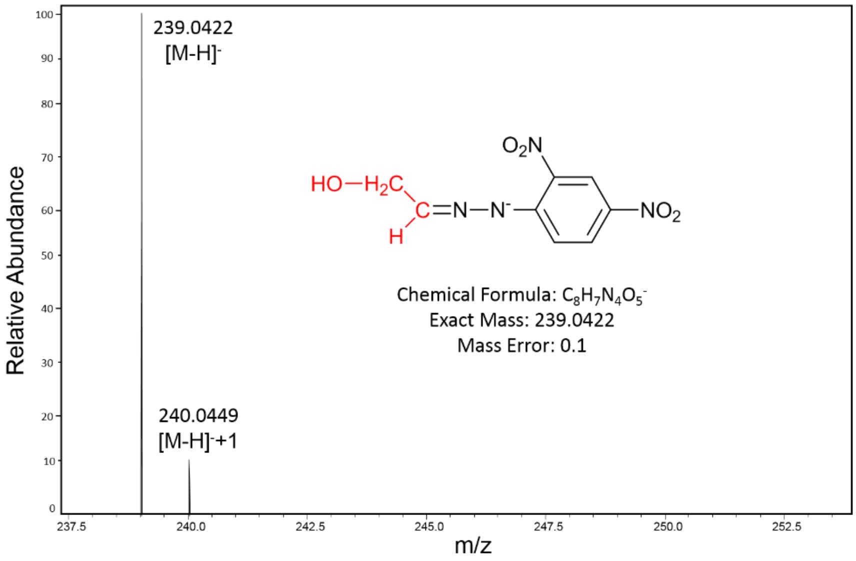

11.4. LC-QTOF analysis of **9a**

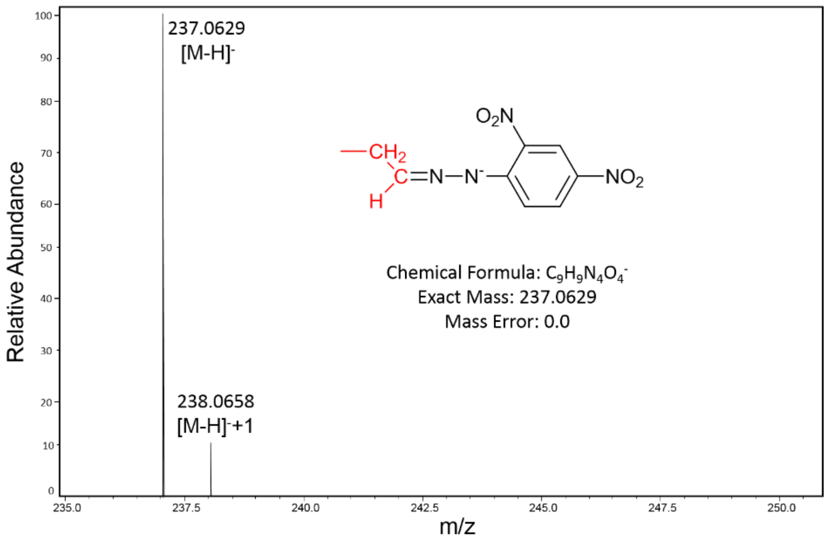

11.5. QTOF analysis of **11a**

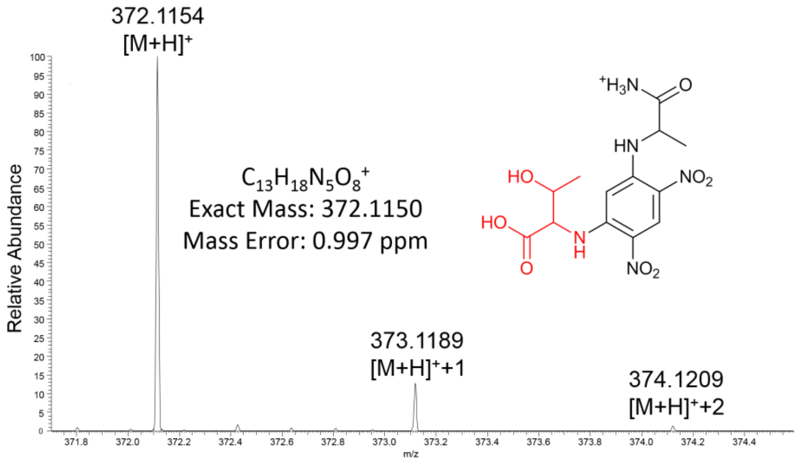

11.6. HPLC-ESI-MRMS analysis of **6a**

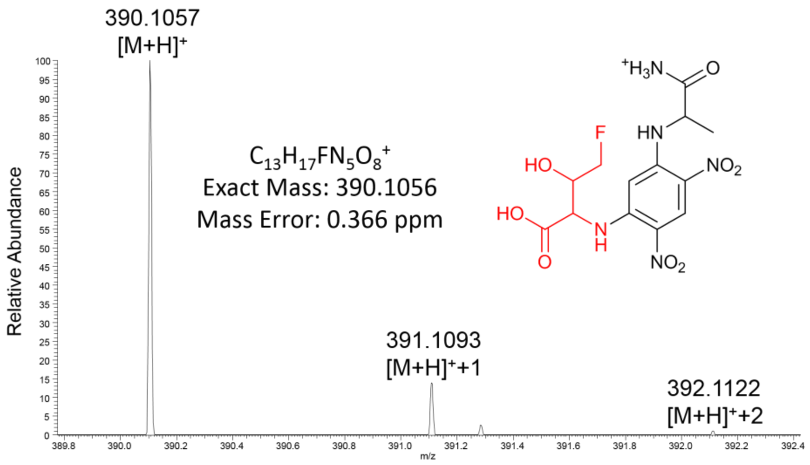

11.7. HPLC-ESI-HRMS analysis of **1a**

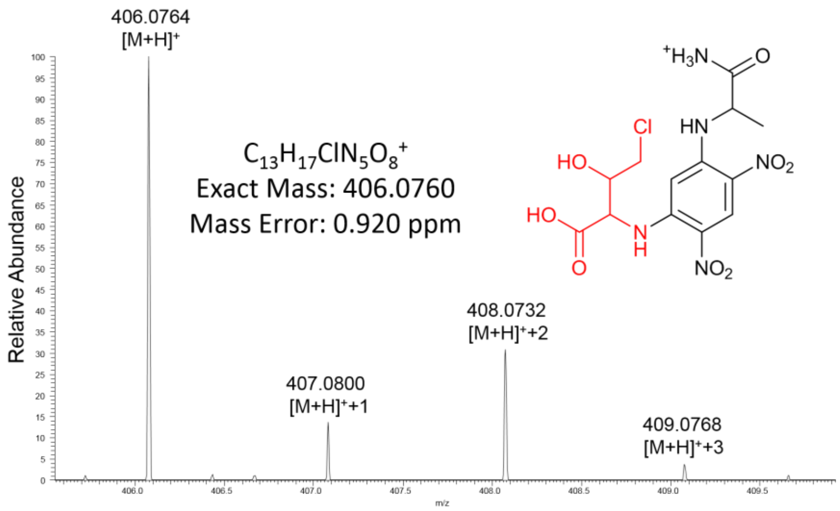

11.8. HPLC-ESI-HRMS analysis of **8a**

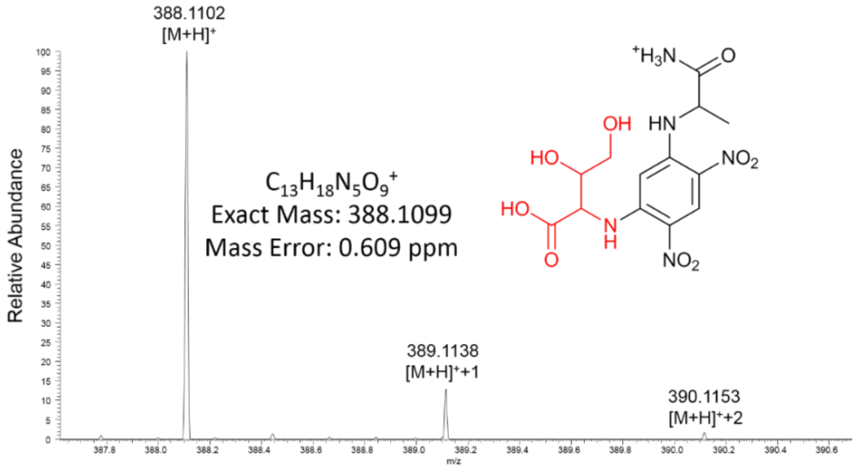

11.9. HPLC-ESI-HRMS analysis of **10a**

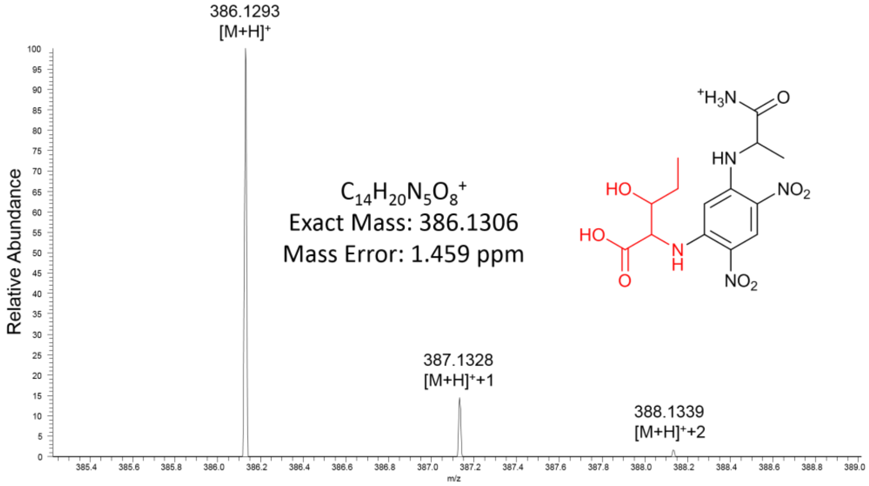

11.10. HPLC-ESI-HRMS analysis of **12a**

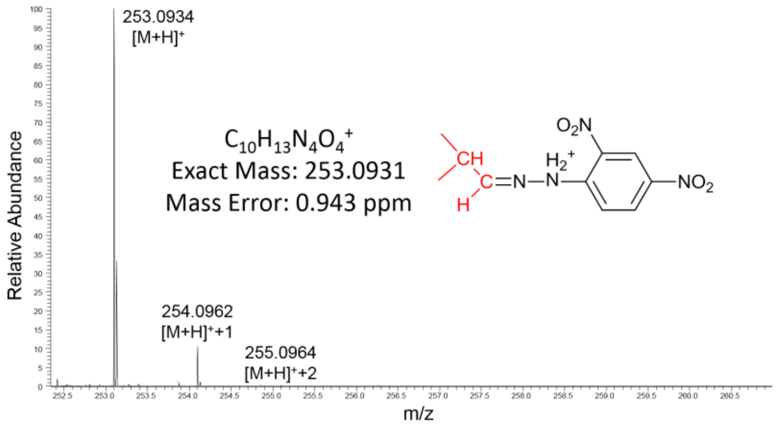

11.11. HPLC-ESI-HRMS analysis of **13a**

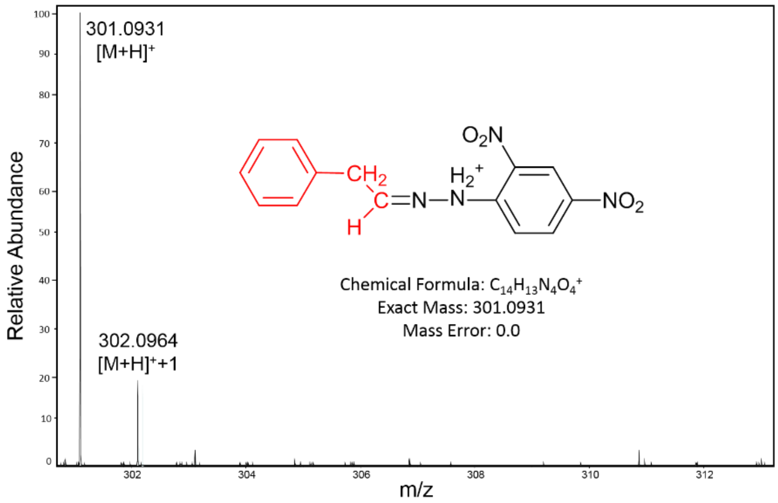

11.12. HPLC-ESI-HRMS analysis of **15a**

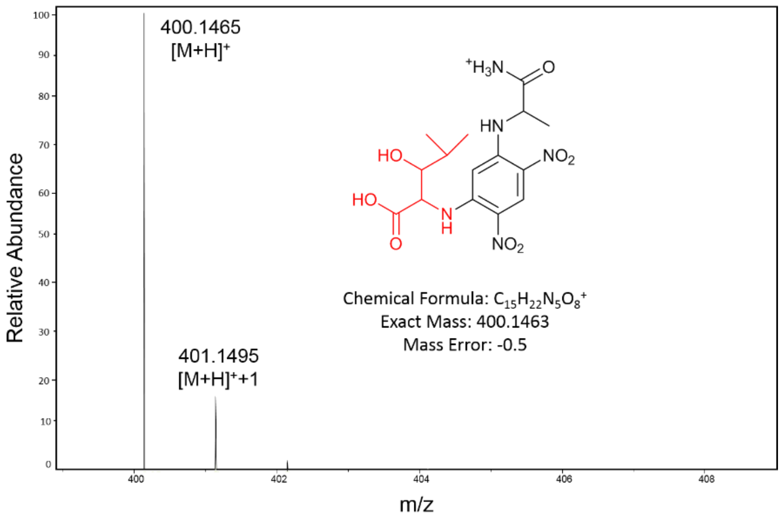

11.13. HPLC-ESI-HRMS analysis of **14a**

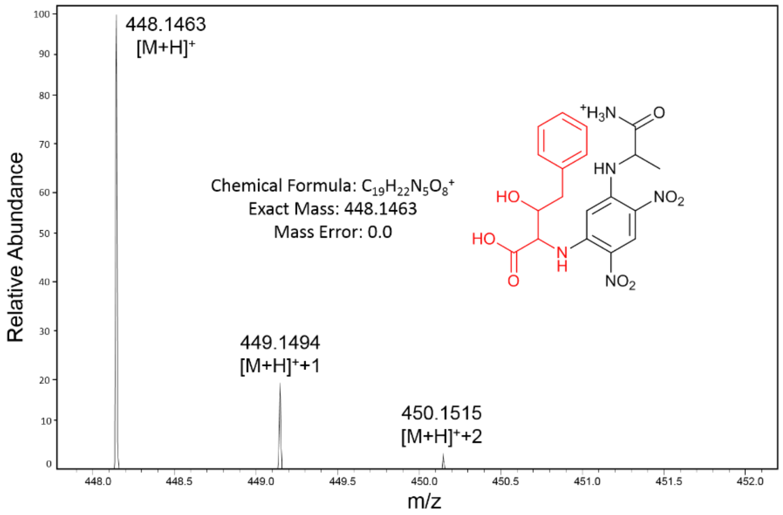

11.14. HPLC-ESI-HRMS analysis of **16a**

Figure S11. The HPLC-ESI-HRMS analysis of substrates and products generated in the FTaseMA assays. (In relation to Table S3)

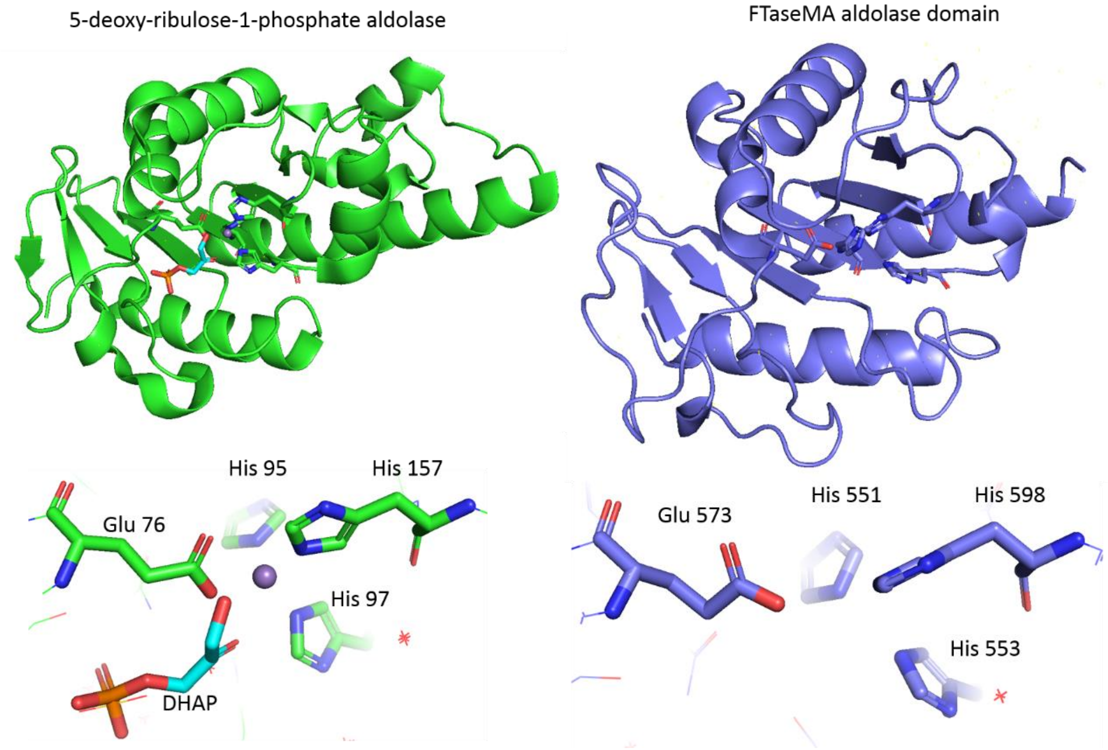

Figure S12. The 3D structure alignment of FTaseMA and 5-deoxy-ribulose-1-phosphate aldolase.

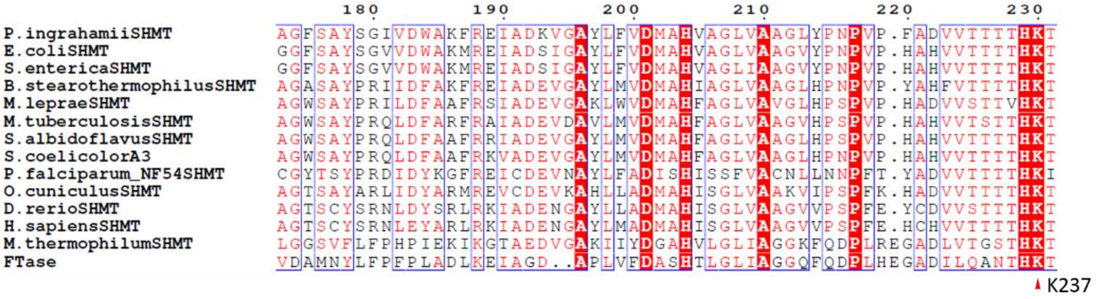

Figure S13A. The multiple sequence alignment of FTaseMA with SHMTs, suggesting that K237 in the first S domain of FTaseMA is the key residue in its active site. The alignment was generated using MEGA7 (1).

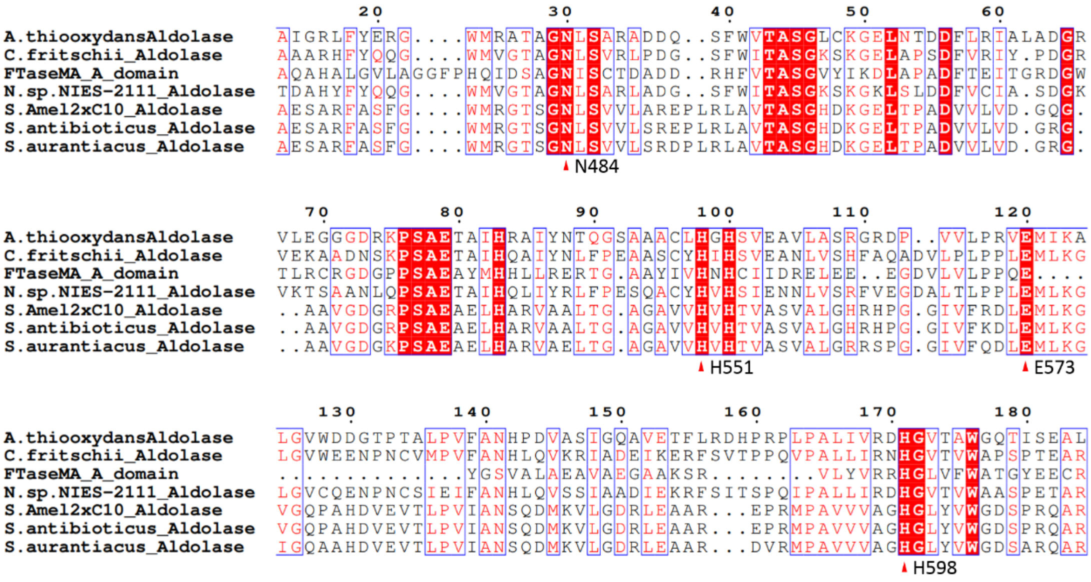

Figure S13B. The multiple sequence alignment of FTaseMA with other Class II metal dependent aldolases, suggesting that the four residues, H551-H553-E573-H598 in FTaseMA numbers, together with the key residue, N484, are highly conserved. The alignment was generated using MEGA7. The mutated amino acids in this study are marked by red triangles. The highlighted conserved amino acids figure was generated using ESPript (2).

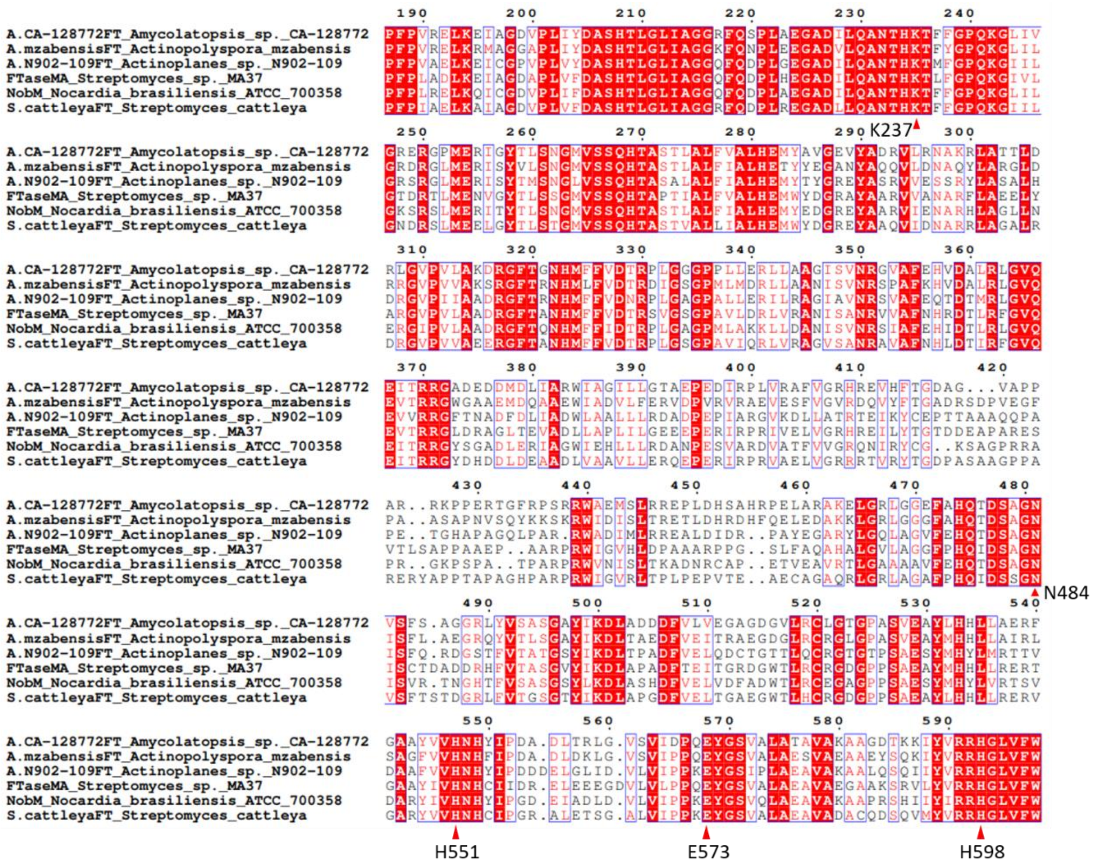

Figure S13C. The multiple sequence alignment of FTaseMA with other FTases identified from putative fluorometabolites producing strains. The alignment was generated using MEGA7. The mutated amino acids in this study are marked by red triangles. The highlighted conserved amino acids figure was generated using ESPript (2).

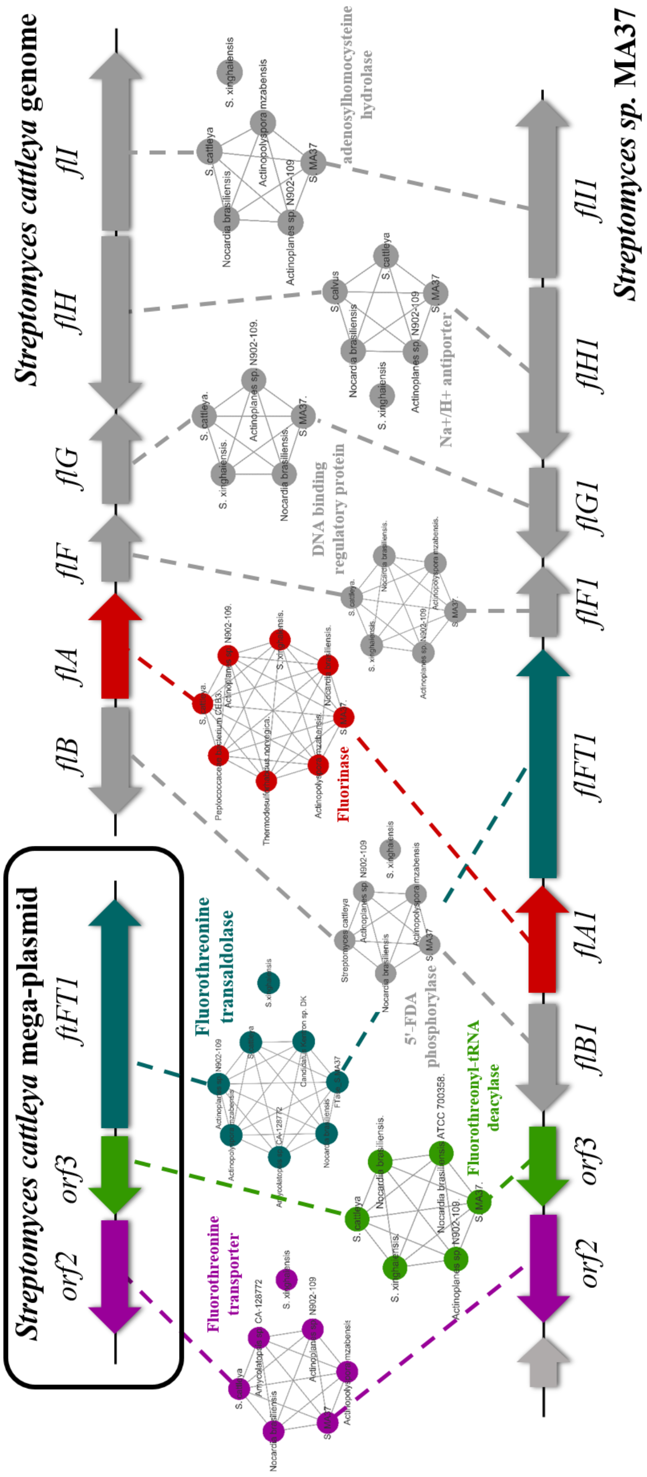

Figure S14. A homologous network of the biosynthetic gene cluster of 4-FT from *Streptomyces* sp. MA37 and *Streptomyces cattleya* constructed by all-to-all BLASTP comparison of sequences found in strains containing essential biosynthetic genes involved in the 4-FT biosynthesis. Each node represents a protein and each edge represents the BLASTP pairwise comparison (E-value < 1E^{-10}) between two protein sequences. The FTases, fluorinases, fluorothreonyl-tRNA deacylases and fluorothreonine transporters are coloured with cyan, red, green and purple, respectively. Other genes in the *fl* clusters are coloured with grey.

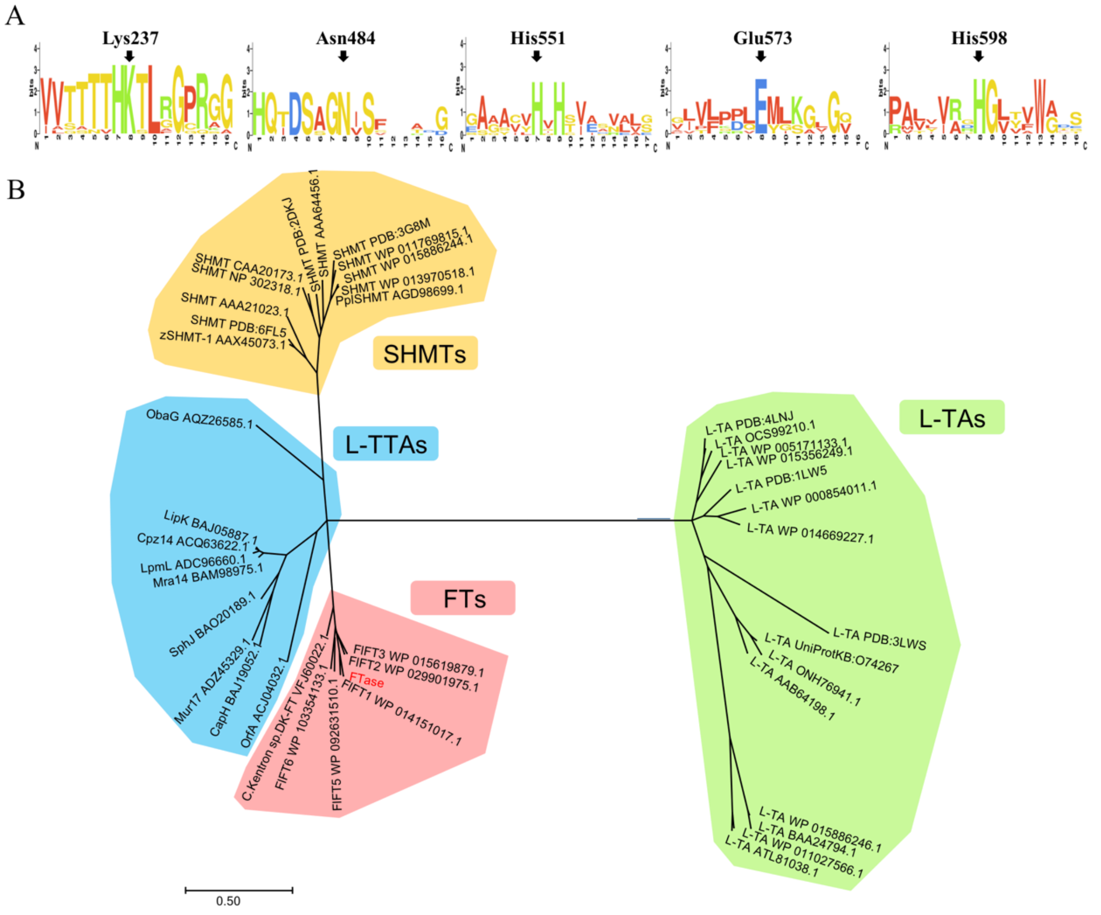

Figure S15. Bioinformatics analysis of FTaseMA active sites and homologs.

(A) Hidden-Markov model logo of active sites identified in FTaseMA. The amino acid residues are indicated by black arrows. **(B)** The phylogenetic analysis of FTases, L-TAs, TTAs and SHMTs. L-TAs, TTAs, SHMTs and FTases with full length sequences are marked with green, blue, orange, and pink backgrounds, respectively. FTaseMA is highlighted in red.

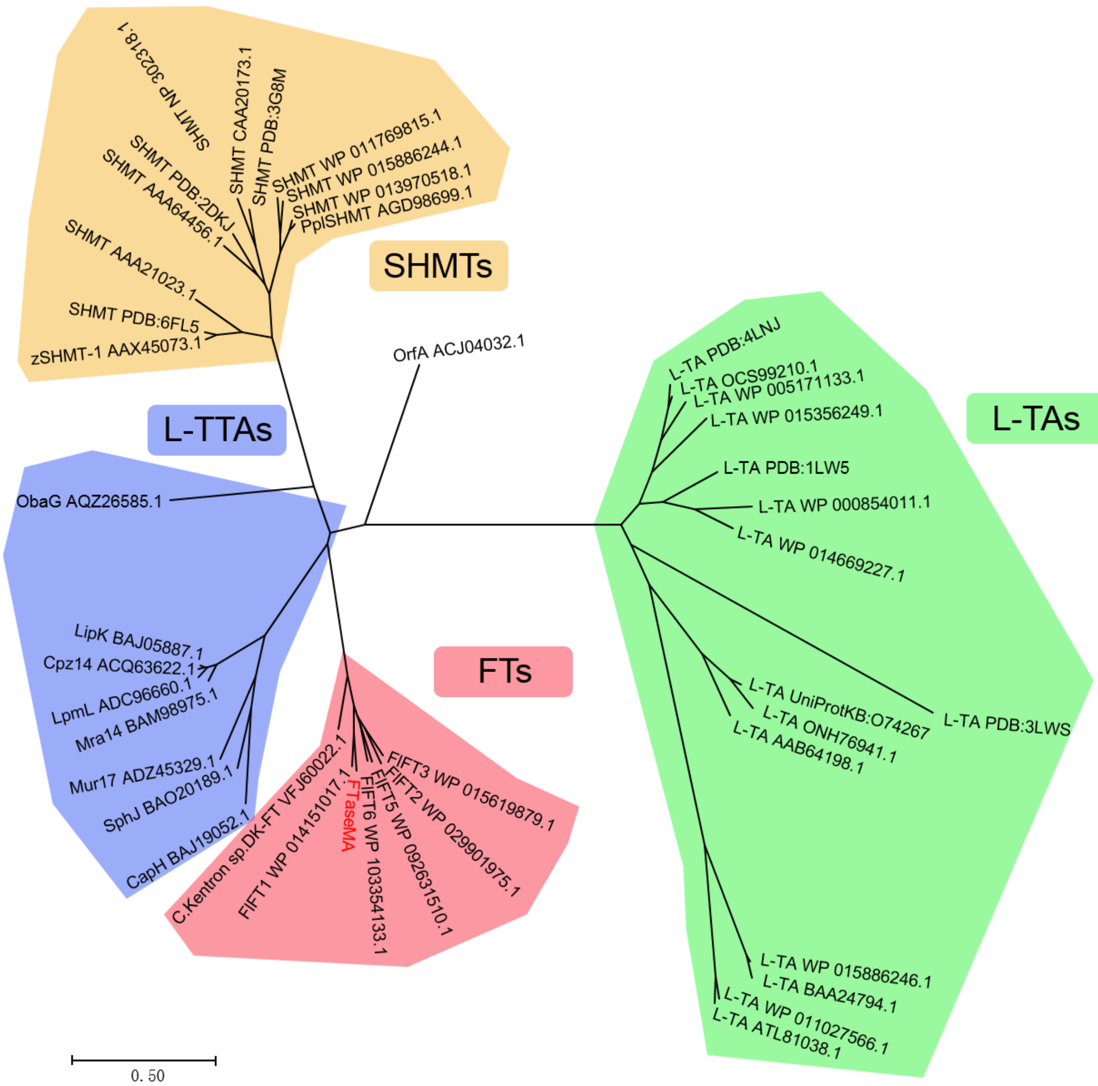

Figure S16. The sequence used to construct the phylogenetic tree was obtained from the National Centre for Biotechnology Information (NCBI)(3) database and the Protein Data Bank (PDB) (4). Sequences were initially aligned with using ClustalW by MEGA7 (1). The A domain of all FTases were manually trimmed based on BLASTP(5) results to avoid interference. The phylogenetic tree was built by MEGA7 using maximum likelihood method with default values.

References

1. Kumar, S., Stecher, G., Li, M., Knyaz, C., and Tamura, K. (2018) MEGA X: molecular evolutionary genetics analysis across computing platforms. *Mol. Biol. Evol.* **35**, 1547-1549
2. Robert, X., and Gouet, P. (2014) Deciphering key features in protein structures with the new ENDscript server. *Nucleic Acids Res.* **42**, W320-W324
3. Benson, D. A., Karsch-Mizrachi, I., Lipman, D. J., Ostell, J., and Sayers, E. W. (2008) GenBank. *Nucleic Acids Res.* **37**, D26-D31
4. Bernstein, F. C., Koetzle, T. F., Williams, G. J., Meyer Jr, E. F., Brice, M. D., Rodgers, J. R., Kennard, O., Shimanouchi, T., and Tasumi, M. (1977) The Protein Data Bank: a computer-based archival file for macromolecular structures. *Journal of molecular biology* **112**, 535-542
5. Altschul, S. F., Gish, W., Miller, W., Myers, E. W., and Lipman, D. J. (1990) Basic local alignment search tool. *Journal of molecular biology* **215**, 403-410
